# Circadian rhythms in glomerular filtration govern natriuresis and diuretic responsiveness

**DOI:** 10.3389/fphys.2026.1828410

**Published:** 2026-06-02

**Authors:** Kaixin Zheng, Anita T. Layton

**Affiliations:** 1 Cheriton School of Computer Science, University of Waterloo, Waterloo, ON, Canada; 2 Departments of Applied Mathematics and Biology, University of Waterloo, Waterloo, ON, Canada; 3 School of Pharmacy, University of Waterloo, Waterloo, ON, Canada

**Keywords:** angiotensin, circadian rhythms, hypertension, kidney, renal transport

## Abstract

Kidney function exhibits circadian rhythms that arise in part from clock-gene–mediated regulation of renal transporter proteins. Key Na^+^ transporters under circadian control include NHE3, SGLT1, NKCC2, NCC, and ENaC, which are targeted by commonly prescribed diuretics. The present study uses computational models of nephron transport to investigate how circadian regulation influences renal function and the natriuretic responses to diuretics in normotensive and Ang II–induced hypertensive rat kidneys. Simulations of conditional Bmal1 knockdown predict marked attenuation of transporter oscillations, segment-specific changes in mean transporter abundance, and a loss of the normal dipping pattern in Na^+^ excretion. In the hypertensive kidney, Na^+^ transport is redistributed from proximal to distal segments, increasing overall natriuresis and diuresis while largely preserving the phase of excretory rhythms when filtration retains its circadian oscillation. Across normotensive and hypertensive conditions, the model predicts that the phase and amplitude of daily excretory rhythms are primarily determined by oscillations in single-nephron glomerular filtration rate (SNGFR). When SNGFR exhibits a normal circadian rhythm (“dippers”), diuretic-induced natriuresis is greatest during the active phase. In contrast, when SNGFR rhythms are absent (“non-dippers”), the time of maximal natriuretic response shifts to the inactive phase, when transporter expression is lowest. Thus, the optimal time of diuretic administration depends on circadian phenotype rather than clock time alone. These findings highlight the importance of renal circadian regulation in determining diuretic responsiveness and suggest that dosing strategies may need to account for individual dipping status.

## 1 Introduction

The kidney is a major regulator of extracellular fluid and whole-body electrolyte homeostasis. In particular, the kidney adjusts the fraction of Na^+^ in its glomerular filtrate that it reabsorbs, to match the amount of Na^+^ excreted in the urine with Na^+^ intake. Given that the kidneys receive 20-25% of the cardiac output (Meltzer, 2013), glomerular filtration rate (GFR) and filtered Na^+^ load are high, and to achieve Na^+^ balance, only about 1% of the filtered Na^+^ is excreted in the urine (in a normal kidney) (Bie, 2018). This necessitates a highly precise adaptation of the renal transport system. Indeed, almost all nephron segments participate in the reabsorption of filtered Na^+^. Along the proximal tubule, the Na^+^/H^+^ exchanger 3 (NHE3) mediates the reabsorption of a large fraction of the filtered Na^+^ (about 50%-70%, more in male rodents) (Dominguez Rieg and Rieg, 2024). The NHE3 also plays a key role in the pressure natriuresis response, whereby an increase in blood pressure leads to an increase in Na^+^ excretion (McDonough et al., 2003). The thick ascending limb is another major Na^+^-reabsorbing segment, where the Na^+^-K^+^-2Cl^-^ cotransporter 2 (NKCC2) on the apical membrane is responsible for 25%-40% of the Na^+^ transport (more in females) (Castrop and Schießl, 2014; Hu et al., 2019). The importance of NKCC2 for Na^+^ balance can be seen in the powerful antihypertensive effect of loop diuretics, which inhibit NKCC2. Among the downstream segments, Na^+^-Cl^-^ cotransport (NCC) on the apical membrane of the distal convoluted tubule mediates Na^+^ uptake, as does the epithelial Na^+^ channel (ENaC) along the principal cells of the connecting tubule and collecting duct (Loffing and Korbmacher, 2009). These segments are responsible for “fine-tuning” the final urinary excretion, and the importance of NCC and ENaC in Na^+^ balance is evinced by the extensive use of thiazide diuretics and K^+^-sparing diuretics, which target NCC and ENaC, respectively, in treatment of hypertension.

The maintenance of fluid and electrolyte homeostasis by the kidney is modulated by multiple hormonal and neural systems, including the renin–angiotensin–aldosterone system (RAAS), renal sympathetic signaling, natriuretic peptides, and vasoactive mediators. Among these, Ang II plays a major role in modulating renal Na^+^ transport at a molecular level. An overactive RAAS may lead to Na^+^ retention, K^+^ loss, and an increase in blood pressure. In particular, angiotensin II (Ang II) regulates renal Na^+^ transport at a molecular level, and chronic infusion of Ang II induces vasoconstriction and anti-natriuresis (Hall, 1986; Benigni et al., 2010). Salt retention is a consequence of the Ang II-induced changes in key renal electrolyte transporters (Nguyen et al., 2013; Giani et al., 2015; Veiras et al., 2020): downregulation of NHE3 and NKCC2 in the proximal tubule and medullary thick ascending limb, upregulation of NKCC2 in the cortical thick ascending limb, NCC, and ENaC. These changes result in a downstream shift in Na^+^ transport capacity of the nephron, Na^+^ and fluid imbalance, and ultimately hypertension (Edwards and McDonough, 2019; Veiras et al., 2020; Edwards et al., 2023).

Describing a primary function of the kidney as to maintain the homeostasis of fluid and electrolyte may give the impression that with sufficient adjustments and feedback control, a steady state or equilibrium can be achieved. However, this picture is incomplete: the kidney, like most physiological systems, exhibits circadian rhythms with a 24-h period. In the mammalian kidney, the circadian rhythms are co-mediated by the central clock, which resides in the suprachiasmatic nucleus (SCN) of the hypothalamus, and by the peripheral clocks within the renal cells that can oscillate independently of the SCN (Honma, 2018). Both the central and peripheral clocks “tick” as a result of the interactions among a network of core clock genes (Leloup and Goldbeter, 2003; Schibler et al., 2015; Woller et al., 2016). Briefly, the basic helix-loop-helix ARNT like 1 (Bmal1) and Clock dimerize to induce the transcription of Period (Per) and Cryptochrome (Cry) genes. The proteins PER and CRY then heterodimerize to act on the protein complex CLOCK-BMAL1, thereby inhibiting their own transcription.

This feedback loop of the core clock genes results in oscillations in protein levels, which drive circadian rhythms observed in kidney function, including the marked reduction in the volume of urine excreted during the night (in diurnal animals) compared to the volume excreted during the day (Stow and Gumz, 2011; Firsov and Bonny, 2018). Similar oscillations are observed in the urinary excretion of electrolytes, renal plasma flow and glomerular filtration rate. The circadian rhythms of key kidney function arise in part from the regulation by clock proteins of renal transporter genes, including those of NHE3, Na^+^-glucose cotransporter 1 (SGLT1), NKCC2, NCC, and ENaC (Stow and Gumz, 2011; Firsov and Bonny, 2018).

As previously noted, NKCC2, NCC, and ENaC are the targets of loop, thiazide, and K^+^-sparing diuretics, common medications for hypertension (Arumugham and Shahin, 2024). Given the diurnal variations in the expression of these transporters, how do the natriuretic and diuretic effects of these diuretics vary during the day? To answer this question, we simulated Na^+^ transporter inhibition using our recently published computational models of water and electrolyte transport along the nephrons of a male kidney that represent the regulation of transporter activities by circadian clock proteins.

## 2 Materials and methods

### 2.1 Modeling the circadian clock network

**Figure 1:**
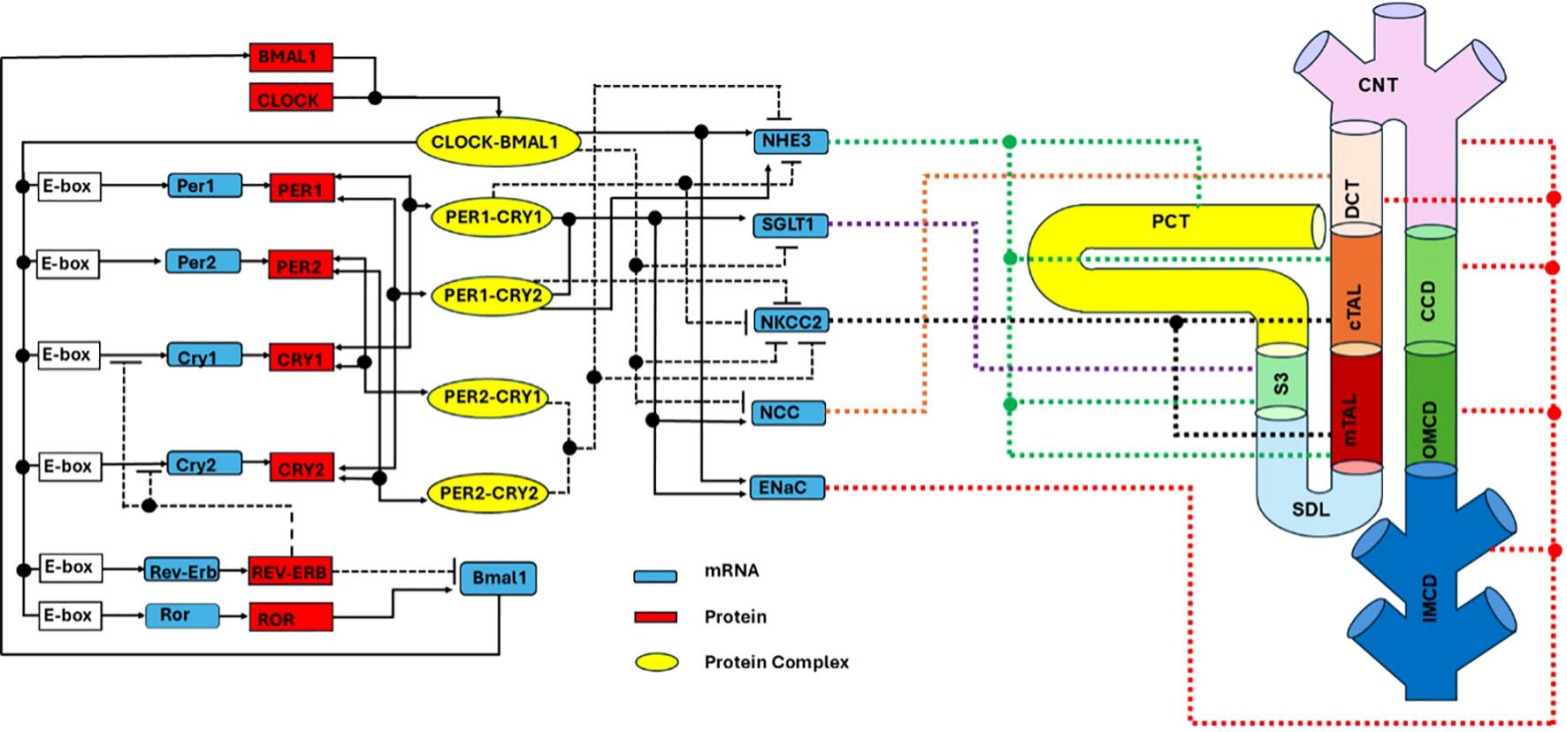
Schematic network of our mathematical model, which describes the interactions of core clock components with key renal transporter genes. In the core clock network, mRNAs are denoted by blue boxes, proteins by red boxes, and protein complexes by yellow ovals. Solid lines represent the transactivation and dotted lines represent inhibition. The model nephron includes the proximal convoluted tubule (PCT), S3 segment, medullary thick ascending limb (mTAL), cortical thick ascending limb (cTAL), distal convoluted tubule (DCT), connecting tubule (CNT), cortical collecting duct (CCD), outer medullary collecting duct (OMCD), and inner medullary collecting duct (IMCD). Renal transporters that are regulated by the circadian clock include NHE3, SGLT1, NKCC2, NCC, and ENaC.

To simulate the modulation of key renal transporter activities in a male rat by the circadian clock, we first develop a mathematical model of the core clock in the male rat kidney. The clock model comprises a number of transcription factors that regulate gene expression: the period homologs Per1 and Per2, the cryptochromes homologs Cry1 and Cry2, Rev-Erb and RAR-related orphan receptor (Ror), brain and muscle ARNT-Like 1 (Bmal1), and circadian locomotor output cycles kaput (CLOCK). A schematic diagram of the clock network is shown in **Figure 1**. Equations for the clock model are given in the Appendix, Eqs. A1-A25. Model parameters were obtained by fitting predicted profiles for mRNA expression levels of core clock genes (Per1, Per2, Cry1, Cry2, Rev-Erb, Ror, Bmal1) with their corresponding measured data reported in Ref (Zhang et al., 2014). and database (Pizarro et al., 2012), obtained for the dark-dark cycles (shown in **Appendix, Tables A1—A7**). Datasets were selected based on kidney specificity, temporal resolution sufficient to resolve circadian dynamics (2–4 h sampling), and experimental protocols designed to isolate intrinsic circadian rhythms (e.g., constant darkness or controlled light–dark cycles). CircaDB (Pizarro et al., 2012) was chosen because it provides curated, normalized, and rhythmically annotated kidney-specific expression profiles derived from Zhang et al. (2014), with dense temporal sampling suitable for model fitting.

### 2.2 Modeling renal epithelial transport and its circadian regulation

The model represents a superficial nephron of a male rat kidney. As depicted in **Figure 1**, the model nephron is divided into 10 functionally distinct segments: proximal convoluted tubule (PCT), proximal straight tubule (S3), short descending limb (SDL), medullary thick ascending limb (mTAL), cortical thick ascending limb (cTAL), distal convoluted tubule (DCT), connecting tubule (CNT), cortical collecting duct (CCD), outer medullary collecting duct (OMCD), and inner medullary collecting duct (IMCD). Each segment is modeled with a series of computational cells assigned with specific physical dimensions, transporter profile, and membrane permeability. The present study focuses on Na^+^ and, to a lesser extent, K^+^, but the model also accounts for Cl^−^, HCO_3_^−^, H_2_CO_3_, CO_2_, NH_3_, NH_4_^+^, HPO_4_^2−^, H_2_PO_4_^−^, H^+^, HCO_2_^−^, H_2_CO_2_, urea, and glucose. In each computational cell, steady-state luminal, cellular, and paracellular concentrations and fluxes are calculated based on water conservation, nonreacting solute conservation, and pH conservation. Model equations can be found in Ref (Layton, 2022).

We represent the circadian regulation of key Na^+^ transporters, including NHE3, expressed on the PCT, S3, mTAL, and cTAL, sodium-glucose cotransporter 1 (SGLT1) on the S3, NKCC2 on the mTAL and cTAL, NCC on the DCT, and ENaC along the DCT, CNT, and CD. Transporter activities are assumed proportional to the corresponding mRNA expression levels, and fluctuate with core clock protein levels. The model does not explicitly represent time-varying circulating hormones such as renin or aldosterone; instead, their downstream effects on transporter regulation are incorporated implicitly through experimentally derived transporter expression profiles and Ang II–dependent remodeling. The links are described in Eqs. A8-A12 and summarized in **Figure 1**. Model parameters were obtained by fitting predicted profiles for the transporters (NHE3, SGLT1, NKCC2, NCC, ENaC) with data reported in Refs (Zhang et al., 2015; Crislip et al., 2020; Layton and Gumz, 2022). These datasets were selected based on the availability of kidney-specific circadian expression profiles for the transporters of interest and adequate temporal resolution. In cases where rat-specific circadian data were limited, mouse datasets were used, consistent with prior studies, under the assumption that key features of circadian regulation are qualitatively conserved across species.

### 2.3 Modeling the circulation regulation of renal hemodynamics

As was done in Ref (Layton and Gumz, 2022), the model represents the single-nephron GFR (SNGFR) of a wild-type male rat in a light-dark cycle, denoted ${\text{SNGFR}}_{WT}$, as a sinusoidal function of the Zeitgeber Time (ZT), denoted t, with a peak at ZT16 as given by Equation 1:

(1) $${\text{ SNGFR}}_{WT}(t)={\text{SNGFR}}_{0}(1+0.12\sin(\frac{2\pi (t-16+6)}{24}))$$

ZT=0 (lights on) marks the start of the rest phase for nocturnal animals, whereas ZT12 (lights off) denotes the start of the active phase. The baseline ${\text{SNGFR}}_{0}$ is taken to be 32 nl/min for the superficial nephron of a male rat.

GFR in Bmal1 knockout mice exhibits an ultradian rhythm different from wild-type, with two peaks at ZT4 and ZT16; the 24-hour cumulative GFR are similar in the two genotypes (Ansermet et al., 2019). Based on these findings, we model SNGFR in Bmal1-knockout rat, denoted ${\text{SNGFR}}_{KO}$, as:

(2) $${\text{SNGFR}}_{KO}(t)={\text{SNGFR}}_{0}(1+0.12\sin(\frac{2\pi (t-4+3)}{12}))\;$$

### 2.4 Modeling the effects of hypertension on renal epithelial transport

We investigated renal function in a male rat model of hypertension induced by a 14-day infusion of Ang II. Following the methodology developed in Ref (Zheng and Layton, 2024)., we incorporated hypertension-associated alterations into the epithelial transport framework described above. These modifications include changes in transporter activities, membrane permeabilities, and interstitial fluid composition that collectively reflect the renal remodeling observed in Ang II–dependent hypertension. Among the transporters for which circadian regulation is explicitly represented in the present model, NHE3 and NKCC2 along the mTAL are downregulated, whereas NKCC2 along the cTAL, NCC, and ENaC are upregulated; SGLT1 expression is assumed to remain unchanged. In the absence of time-resolved experimental data characterizing circadian regulation under hypertensive conditions, the coupling between clock proteins and transporter activities is assumed to be preserved, and the relative circadian temporal profiles are taken to be the same as in normotension. Hypertension is therefore represented through changes in baseline transporter abundance, membrane properties, and interstitial conditions, rather than through modifications to circadian phase or waveform. This assumption reflects a parsimonious modeling choice that isolates the effects of baseline regulatory remodeling, while acknowledging that hypertension may in reality alter circadian timing, which is not captured here.

### 2.5 Modeling the effects of diuretics on renal epithelial transport

We consider the effects of loop diuretics, thiazide diuretics, and K^+^-sparing diuretics on renal transport and excretions. We simulate a dose of loop diuretics that induces 70% inhibition of NKCC2, as well as its effect on the kidney’s ability to generate an axial osmolality gradient. To that end, we lower the interstitial fluid concentrations of selected solutes (Layton et al., 2016), but keep the interstitial urea concentration at the papillary at the baseline hypertension value (which is assumed to be lower than in normotension) (Zheng and Layton, 2024). SNGFR is assumed to retain its baseline profile.

Thiazide diuretics inhibit NCC. We simulate a dose of thiazide diuretics that induces 100% inhibition of NCC. All other model parameters remain at baseline values. Interstitial concentration profiles are assumed unchanged.

K^+^-sparing diuretics inhibit ENaC. We simulate a dose of K^+^-sparing diuretics that induces 100% inhibition of ENaC. Interstitial concentration profiles are again assumed unchanged.

## 3 Results

### 3.1 Model predicts diurnal oscillations of clock gene and transporter expression levels

**Figure 2:**
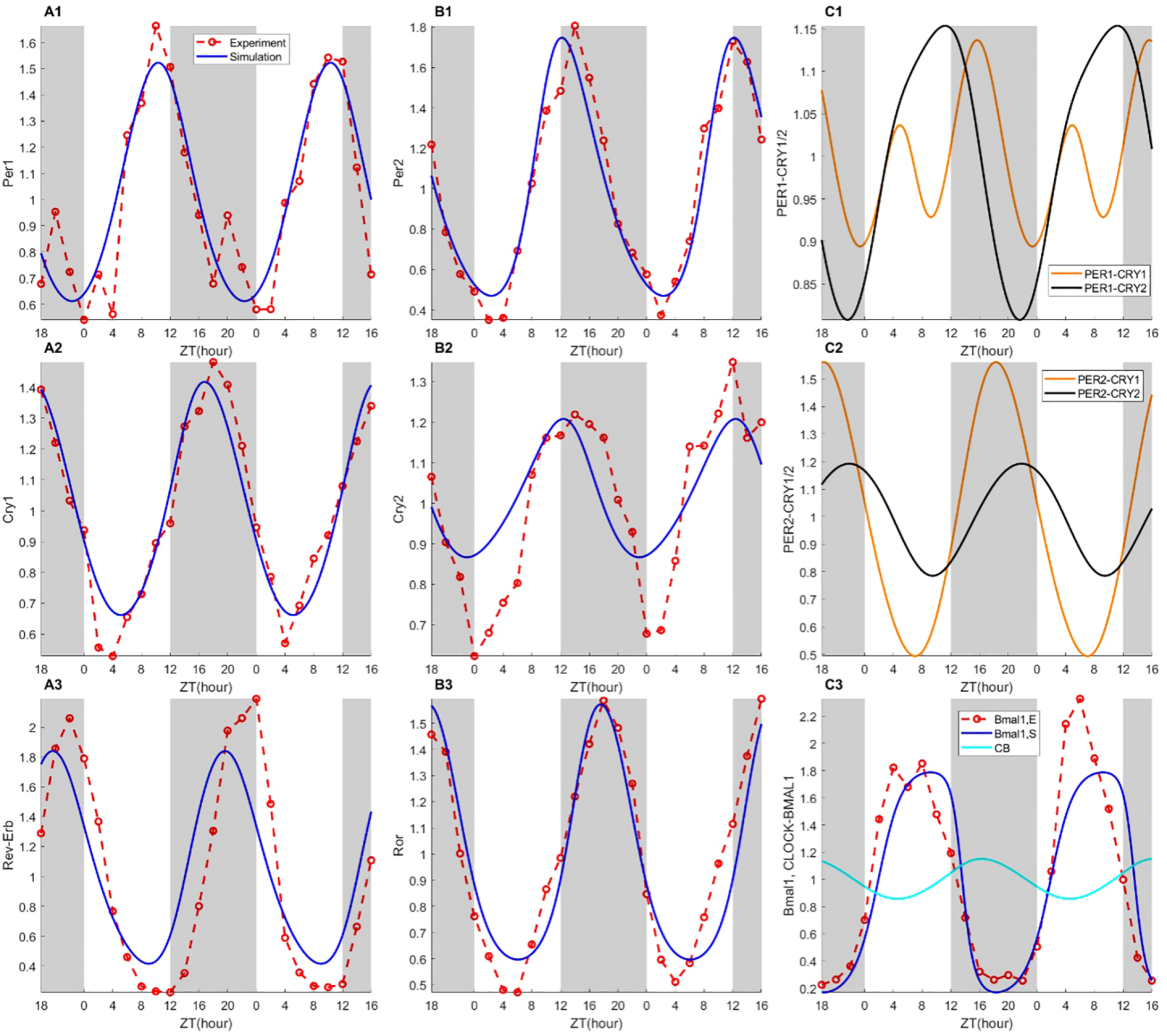
Comparison of simulated circadian gene and protein profiles with data from 46-hour experiments starting at ZT18 (Pizarro et al., 2012; Zhang et al., 2014). **(A1-A3, B1-B3)**, simulated mRNA expression levels (Simulation) compared with experimental data (Experiment), normalized by mean experimental values; **(C1-C2)**, simulated concentrations of PER1–CRY1, PER1–CRY2, PER2–CRY1, and PER2–CRY2 protein complexes, normalized by their respective mean simulated values; **(C3)**, simulated BMAL1 mRNA expression levels (Bmal1, S), experimental BMAL1 mRNA levels (Bmal1, E), and normalized CLOCK–BMAL1 complex concentrations (CB). Gray shading and white regions correspond to dark and light phase, respectively.

**Figure 3:**
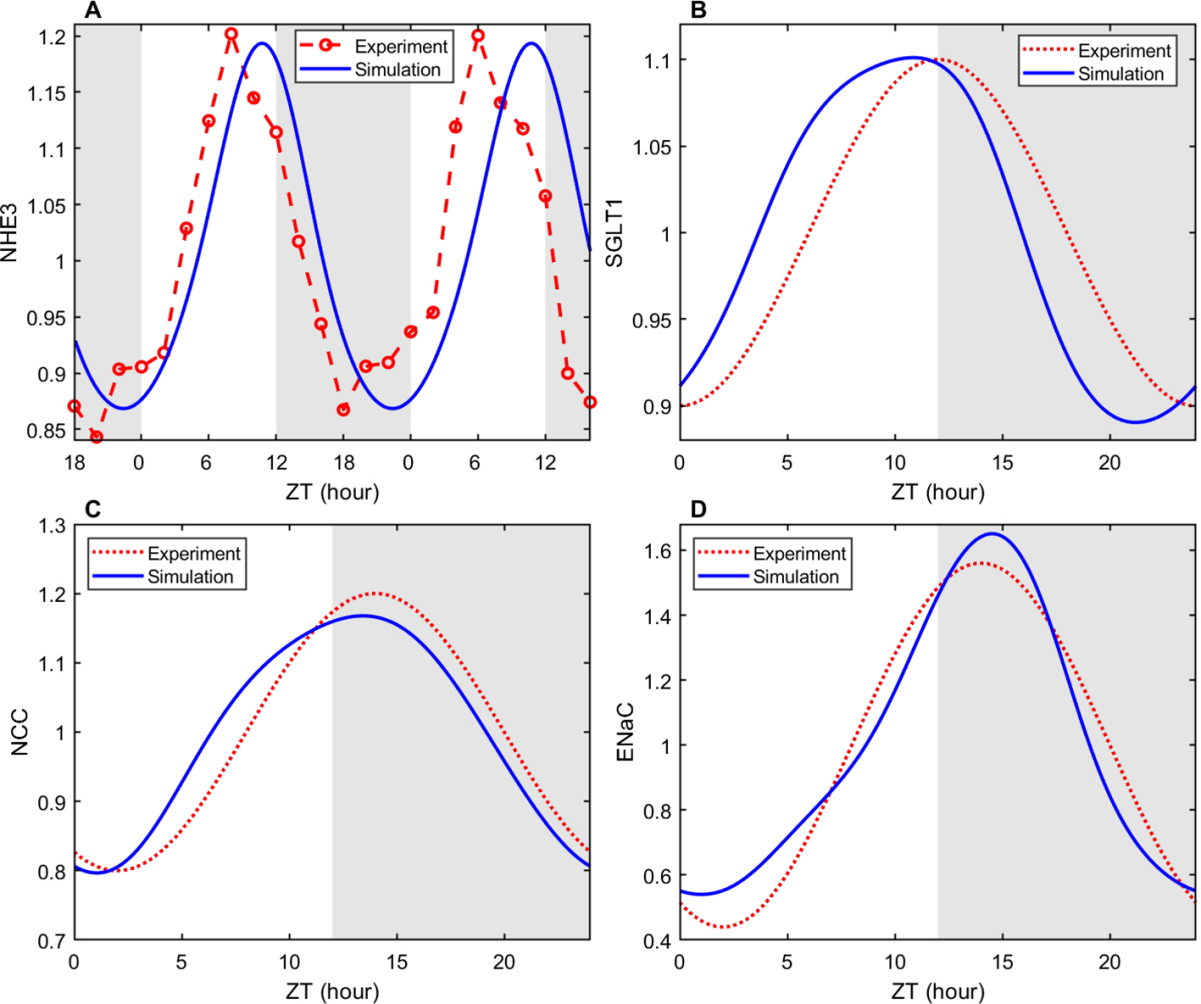
Comparison of simulated transporter expression with corresponding experimental data. **(A)**, NHE3; **(B)**, SGLT1; **(C)**, NCC; **(D)**, ENaC respectively. Experimental profiles in **(B-D)** are sinusoidal curves fitted to the peak times and amplitudes taken from Ref (Zhang et al., 2015).

**Figure 4:**
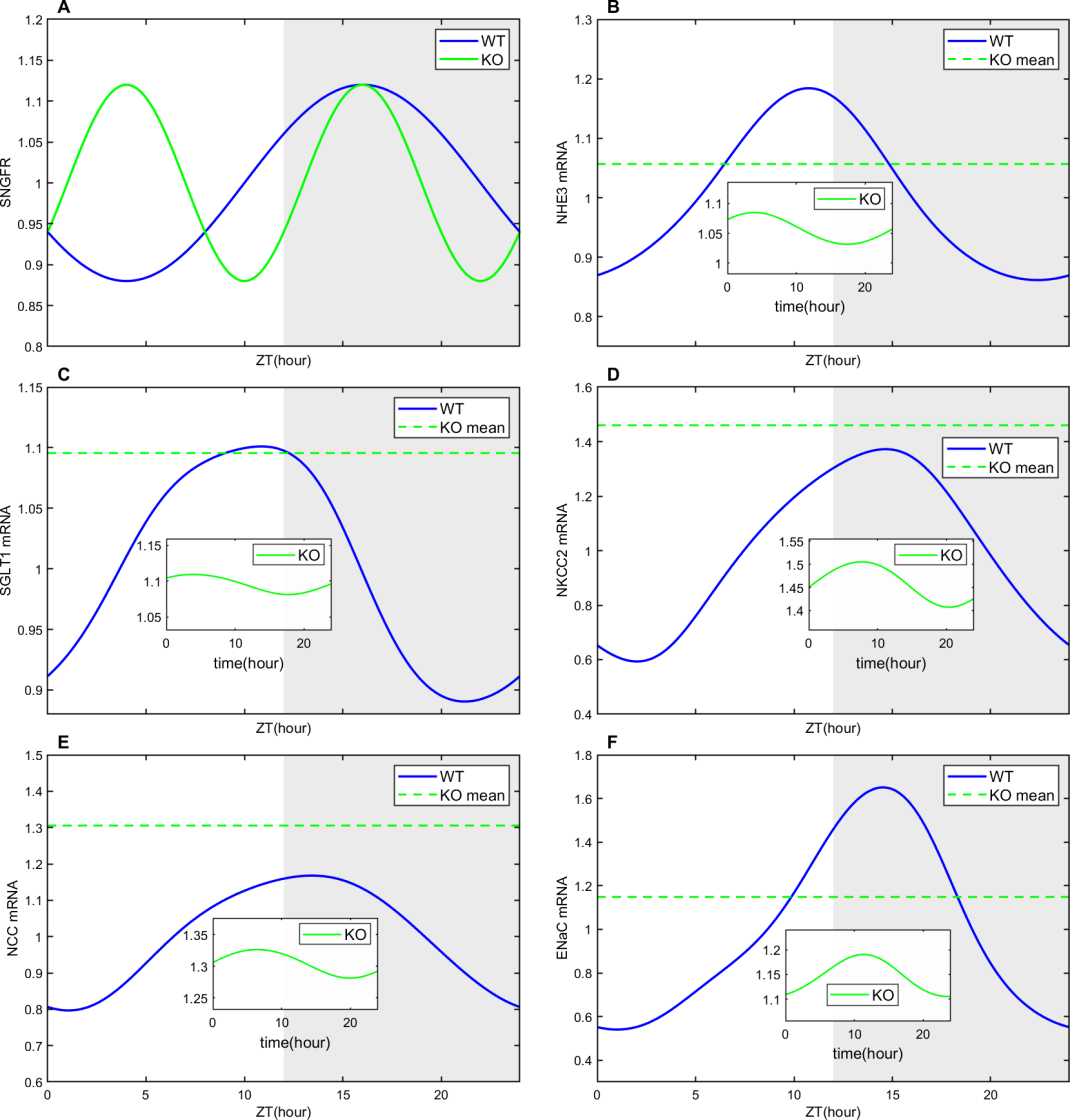
Comparison of model GFR and simulated transporter expression between wild type (WT) and conditional Bmal1 knockdown (KD) male rat. **(A)**, GFR is normalized by baseline GFR at ZT10 in the wild type; **(B-F)** simulated transporter expression levels are normalized by mean experimental values the same as in **Figure 3**. Panel **(D)** shows NKCC2 expression in wild-type (* WT, experimental) and in Bmal1 knockout (Δ KD, experimental, in inset) male rats. Gray shading and white regions correspond to the dark and light phases, respectively.

After parameter fitting, we first assess whether model predictions are physiologically reasonable. Using the baseline model parameters (**Appendix, Tables A1–A7**), the circadian clock network model predicts that the expression levels of all core clock components exhibit limit-cycle oscillations with a 24-h period. Time-profiles of core clock components, together with the experimental data (Zhang et al., 2014), are shown in **Figure 2**. Oscillations in the core clock components drive oscillations in the expression levels of NHE3, SGLT1, NKCC2, NCC, and ENaC; see **Figures 3** and **4D**. Model parameters were chosen to ensure good agreement between the predicted profiles and data. Experimental data for NHE3 and NKCC2 are taken from Refs (Zhang et al., 2014). The experimental profiles for SGLT1, NCC, and ENaC are sinusoidal curves fitted to the peak times and amplitudes taken from or approximated by Ref (Zhang et al., 2015). The predicted oscillatory characteristics (amplitudes and peaks) for all components are summarized in **Table 1**.

**Table 1:** Peak time and relative amplitude of predicted mRNA oscillations in the kidneys of wild-type normotensive male rats.

mRNA	Peak time (ZT)	Relative amplitude
Per1	10	91%
Per2	12	127%
Cry1	17	76%
Cry2	12	34%
Rev-Erb	20	140%
Ror	18	98%
Bmal1	10	160%
NHE3	11	32%
NKCC2	15	64%
SGLT1	11	21%
NCC	14	31%
ENaC	15	110%

Relative amplitude is calculated as (peak - trough)/mean.

### 3.2 Bmal1 knockdown has differential impact on transporter abundance

We next simulate a reduction in Bmal1 activity to represent conditional Bmal1 disruption. In the model, this is implemented by reducing the maximal transcription rate of Bmal1 by 40%, which markedly attenuates the circadian oscillation amplitude of the CLOCK–BMAL1 complex (results not shown). We refer to this condition as a *Bmal1 knockdown* in the model.

Notably, when Bmal1 transcription is completely suppressed in the model, circadian oscillations in both clock components and downstream transporters are abolished. In contrast, experimental studies of conditional Bmal1 knockout indicate that rhythmicity is attenuated but not eliminated, and that SNGFR exhibits altered (e.g., ultradian) oscillatory patterns (Equation 2) (Ansermet et al., 2019). This discrepancy suggests that additional regulatory mechanisms not represented in the present model may sustain oscillatory behavior *in vivo*. The partial reduction in Bmal1 activity adopted here therefore provides a phenomenological representation that preserves oscillatory dynamics while capturing key features of the experimentally observed phenotype.

With suppression of the renal circadian clock, the oscillation amplitudes of key transporter abundances are markedly reduced (**Figure 4**). For example, the model predicts an 85% decrease in the amplitude of NKCC2 oscillations relative to wild type, in agreement with experimental observations. At the same time, the mean NKCC2 expression level is predicted to increase by 46%, also consistent with available data (Crislip et al., 2020). In contrast to the uniform reduction in oscillatory amplitude, the effects on mean transporter abundance are segment specific. The model predicts increased mean expression of SGLT1, NKCC2, and NCC (Crislip et al., 2020), whereas mean NHE3 and ENaC levels remain close to wild-type values. Notably, the transporters that exhibit elevated mean abundance are load responsive: SGLT1 is regulated by filtered glucose delivery, NKCC2 by NaCl delivery to the thick ascending limb, and NCC by distal NaCl load and aldosterone signaling. Thus, when circadian rhythms in SNGFR are abolished, filtered load is redistributed over the day. In the model, the observed increase in mean expression of load-responsive transporters reflects the combined effects of altered filtration dynamics and intrinsic circadian regulation of transporter expression.

In contrast, mean NHE3 and ENaC expression levels remain close to wild type. Indeed, when the maximal Bmal1 transcription rate is further reduced to zero, oscillations for all transporters disappear, with the mean expressions of NHE3 and ENaC reduced by 24% and 65%, respectively, relative to wild type. NHE3 is highly sensitive to luminal flow and pH, whereas ENaC, which controls the final Na^+^ balance, is kept under strict aldosterone-K^+^ balance control, and is indeed often downregulated when upstream Na^+^ reabsorption increases.

**Figure 5:**
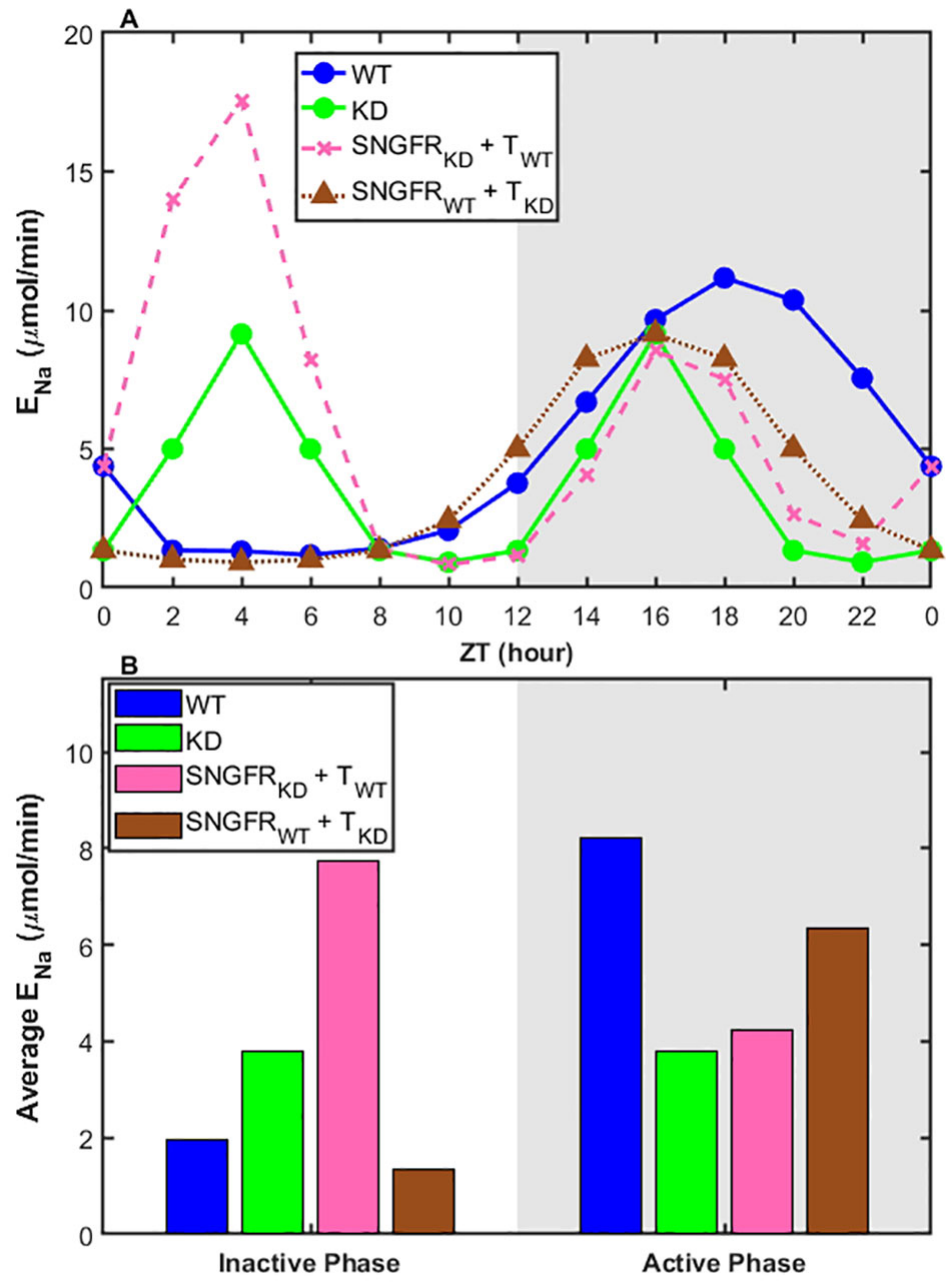
Comparison of predicted urinary Na^+^ excretion (E_Na_) in wild type (WT), Bmal1 knockdown (KD), WT model with Bmal1-KO SNGFR profile (SNGFR_KO_ + T_WT_), WT model with Bmal1-KD transporter levels (SNGFR_WT_ + T_KO_). **(A)**, time profiles. **(B)**, 24-hour averages.

In the Bmal1 knockdown model, the switch from circadian to ultradian rhythms in SNGFR, together with the differential regulation of Na^+^ transporter abundance, yields urinary Na^+^ excretion that exhibits two peaks (**Figure 5A**, green curve). During the inactive (light) phase, 12-hour cumulative Na^+^ excretion is 110% higher in knockdown rats compared to wild type (**Figure 5B**). This is associated with a 7% elevation in SNGFR and the consequent increase in filtered Na^+^ load. Conversely, during the active (dark) phase, Na^+^ excretion is 55% lower (**Figure 5B**), coinciding with a 6% reduction in SNGFR and a 10% increase in cumulative NHE3 activity. This predicted loss of the dipping pattern in excretion aligns with experimental findings in Bmal1-knockout male mice (Johnston et al., 2020), where Na^+^ excretion was elevated by 46% during the inactive phase but reduced by 24% during the active phase.

In summary, the Bmal1 knockdown model markedly attenuates circadian oscillations in transporter abundance while producing segment-specific changes in mean expression. Load-responsive transporters such as SGLT1, NKCC2, and NCC exhibit increased mean abundance, whereas NHE3 and ENaC remain near wild-type levels unless Bmal1 transcription is fully suppressed. Together with the shift from circadian to ultradian SNGFR rhythms, these factors are associated with a redistribution of Na^+^ excretion over the day, increasing excretion during the inactive phase and decreasing it during the active phase, thereby eliminating the normal dipping pattern. Thus, the present formulation should be interpreted as a model of partial clock disruption calibrated to reproduce key experimental features, rather than a direct representation of complete gene knockout.

### 3.3 Transporter regulation attenuates SNGFR-driven natriuresis in Bmal1 knockdown

An examination of the predicted urinary Na^+^ excretion in the knockdown suggests that it is primarily driven by SNGFR, in that both peak at ZT4 and ZT16 and there is little evidence of the oscillations in transporter activities. To reveal any impact of the differential regulation of transporter activities discussed above, we conduct two simulations: one model with wild-type circadian SNGFR and knockdown transporter profiles (“SNGFR_WT_ + T_KO_”), another model with knockdown ultradian SNGFR and wild-type transporter profiles (“SNGFR_KO_ + T_WT_”).

The predicted urinary Na^+^ excretions for these two cases are shown in **Figure 5** and can be compared against the baseline wild-type and full Bmal1-knockdown results. These results suggest that transporter abundance is altered in the knockdown to essentially function as a buffer against SNGFR-driven natriuresis. During the inactive phase when SNGFR is elevated compared to wild-type levels, the upregulated SGLT1, NKCC2, and NCC together increase overall Na^+^ reabsorption and limits Na^+^ excretion (**Figure 5B**). As shown in **Figure 5A**, in the absence of this transporter buffering (i.e., the SNGFR_KO_ + T_WT_ case), Na^+^ excretion would be double that of the full Bmal1-knockdown model at ZT4. The effect of the transporter regulation is less significant during the active phase.

### 3.4 Predicted segmental transport and urinary excretions in normotension and hypertension

**Figure 6:**
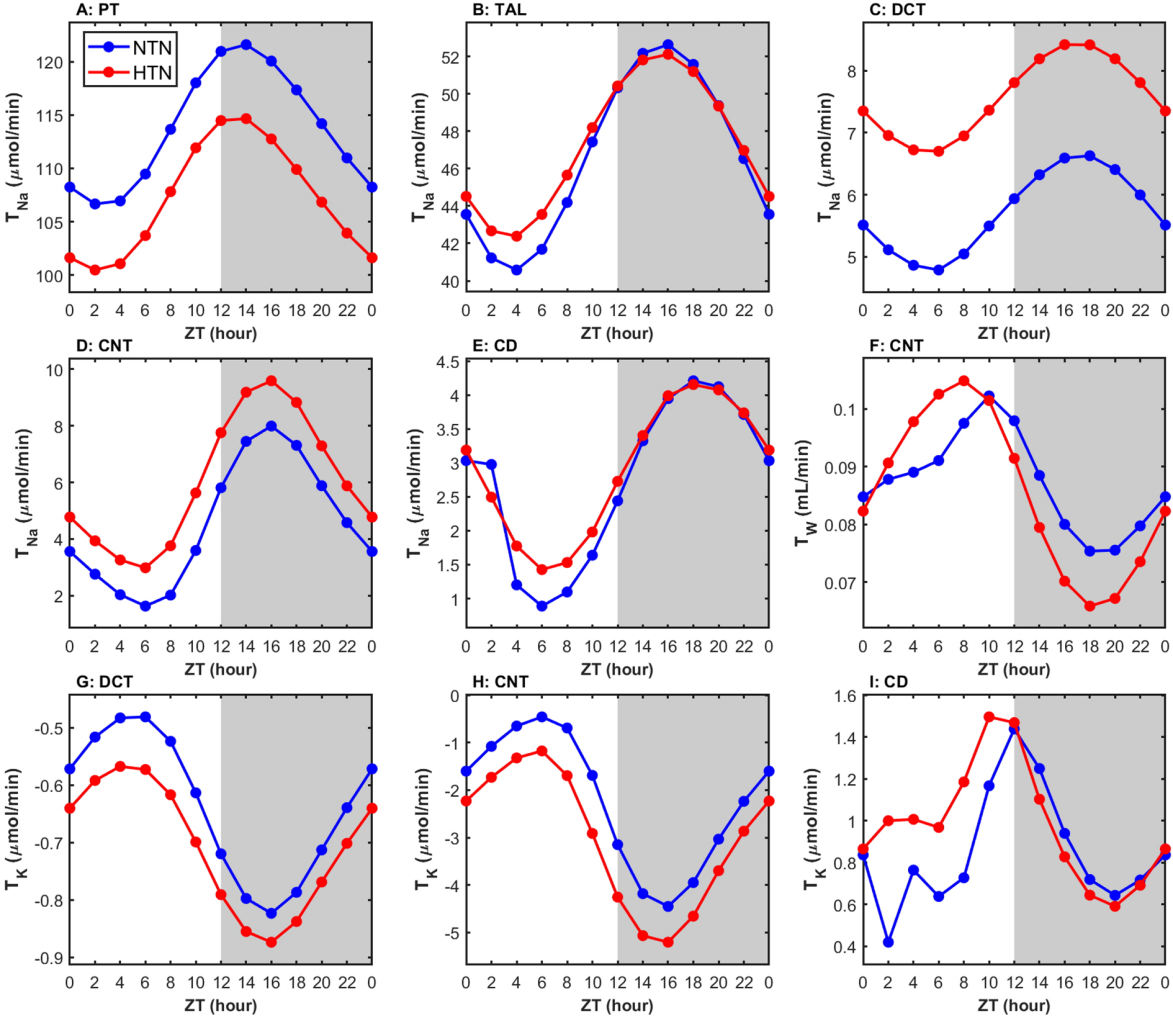
Comparison of segmental Na^+^, K^+^, and water transport in normotension (NTN) and hypertension (HTN). PT, proximal tubule; TAL, thick ascending limb; DCT, distal convoluted tubule; CNT, connecting tubule; CD, collecting duct. T_Na_, Na^+^ transport; T_K_, K^+^ transport; T_W_, water transport.

**Figure 7:**
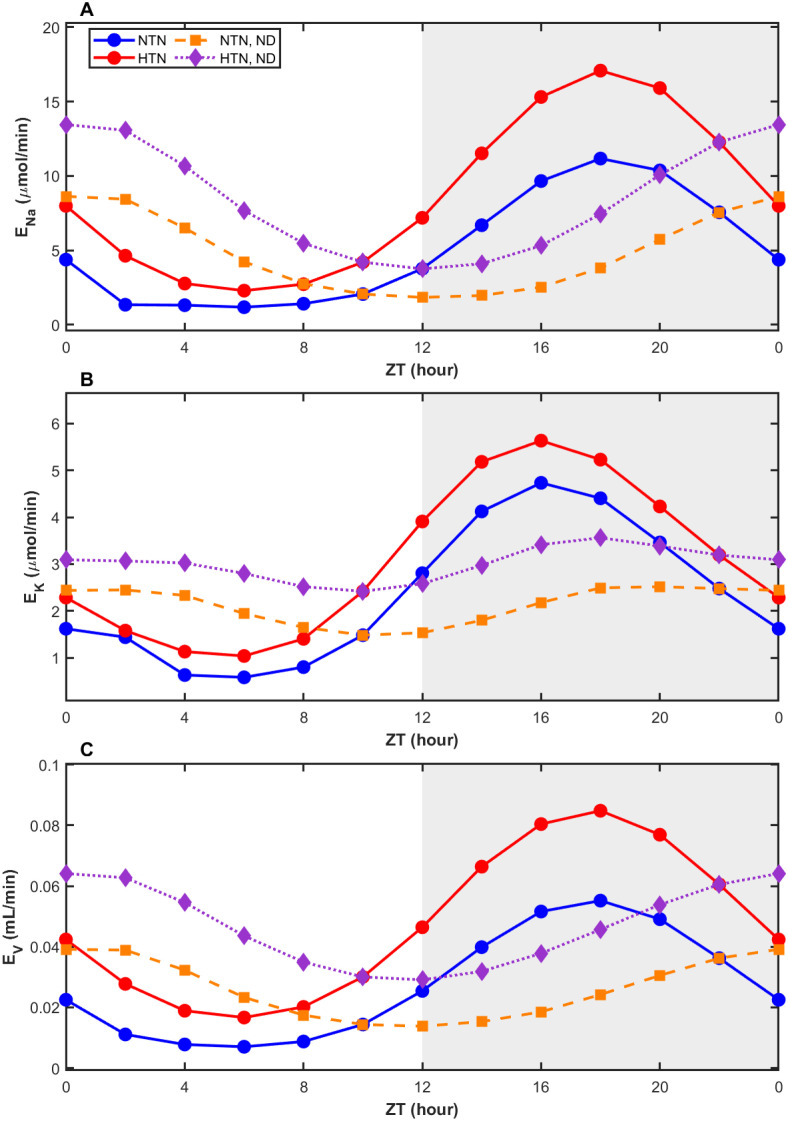
Comparison of urinary Na^+^ excretion [**(A)**, E_Na_], urinary K^+^ excretion [**(B)**, E_K_], and urine output [**(C)**, E_V_] in normotension (NTN), hypertension (HTN), normotension with constant SNGFR (NTN,ND), and hypertension with constant SNGFR (HTN,ND).

In the next set of simulations, we assess the differences in nephron function in a normotensive and hypertensive rat throughout the day. Simulations were conducted for a normotensive and a hypertensive rat kidney, using the same SNGFR and clock gene profiles. Selected predicted segmental transport profiles are shown in **Figure 6**. The predicted filtered Na^+^ load, urinary output, Na^+^ and K^+^ excretions are shown in **Figure 7** (blue and red solid curves).

In the hypertensive model, the downregulation of NHE3 reduces Na^+^ reabsorption along the proximal tubule (**Figure 6A**), shifting the Na^+^ load downstream. Interestingly, Na^+^ transport along the thick ascending limb remains largely unchanged, as the hypertension-induced downregulation in the medullary segment is compensated by upregulation in the cortical segment (**Figure 6B**). Consequently, the increased load is handled by the distal nephron, where Na^+^ reabsorption is enhanced by the upregulation of NCC and ENaC (**Figure 6C**).

Despite these segmental changes, the model predicts no significant phase shift in the urinary excretion profiles. In both normotensive and hypertensive simulations, urinary Na^+^ and volume peak at ZT18, primarily driven by the circadian rhythm of SNGFR (**Figure 7**). However, hypertension significantly elevates urine output and excretion rates throughout the 24-hour cycle. These predicted patterns of natriuresis and diuresis, with increased magnitude but preserved phase, align with experimental reports for hypertensive mice (Douma et al., 2022).

### 3.5 SNGFR oscillations dictate the phase and amplify the amplitude of natriuresis in hypertension

In Ang II-induced hypertension, Na^+^ transporters are differentially regulated such that Na^+^ reabsorption is shifted to distal segments. Direct 24-hour measurements of SNGFR in hypertensive models remain limited, but clinical and experimental studies indicate that hypertension is frequently associated with blunted or phase-shifted circadian patterns of renal hemodynamics, filtration, and sodium handling (Costello et al., 2022; Mohandas et al., 2022). To assess how these changes affect segmental transport and excretion, we conduct simulations of a baseline normotensive (NTN) model, a hypertensive model with baseline SNGFR and hypertension-regulated transporter pattern (HTN), and, to assess the effect of SNGFR oscillations, the “non-dipping” NTN and HTN models with constant SNGFR (NTN-ND and HTN-ND).

The predicted 24-hour profiles of Na^+^ excretion, K^+^ excretion, and urine output for the four cases are shown in **Figure 7**. For the dippers, the NTN and HTN cases exhibit similar circadian patterns that align closely with SNGFR oscillations, peaking at ZT4 and ZT16. The same is true for the non-dippers, except that, in the absence of SNGFR circadian rhythms, the oscillations are now driven by the circadian rhythms in the transporter abundance, and the peaks are shifted to ZT0.

### 3.6 Natriuretic effects of diuretics depend on administration time and blood pressure dipping status

**Figure 8:**
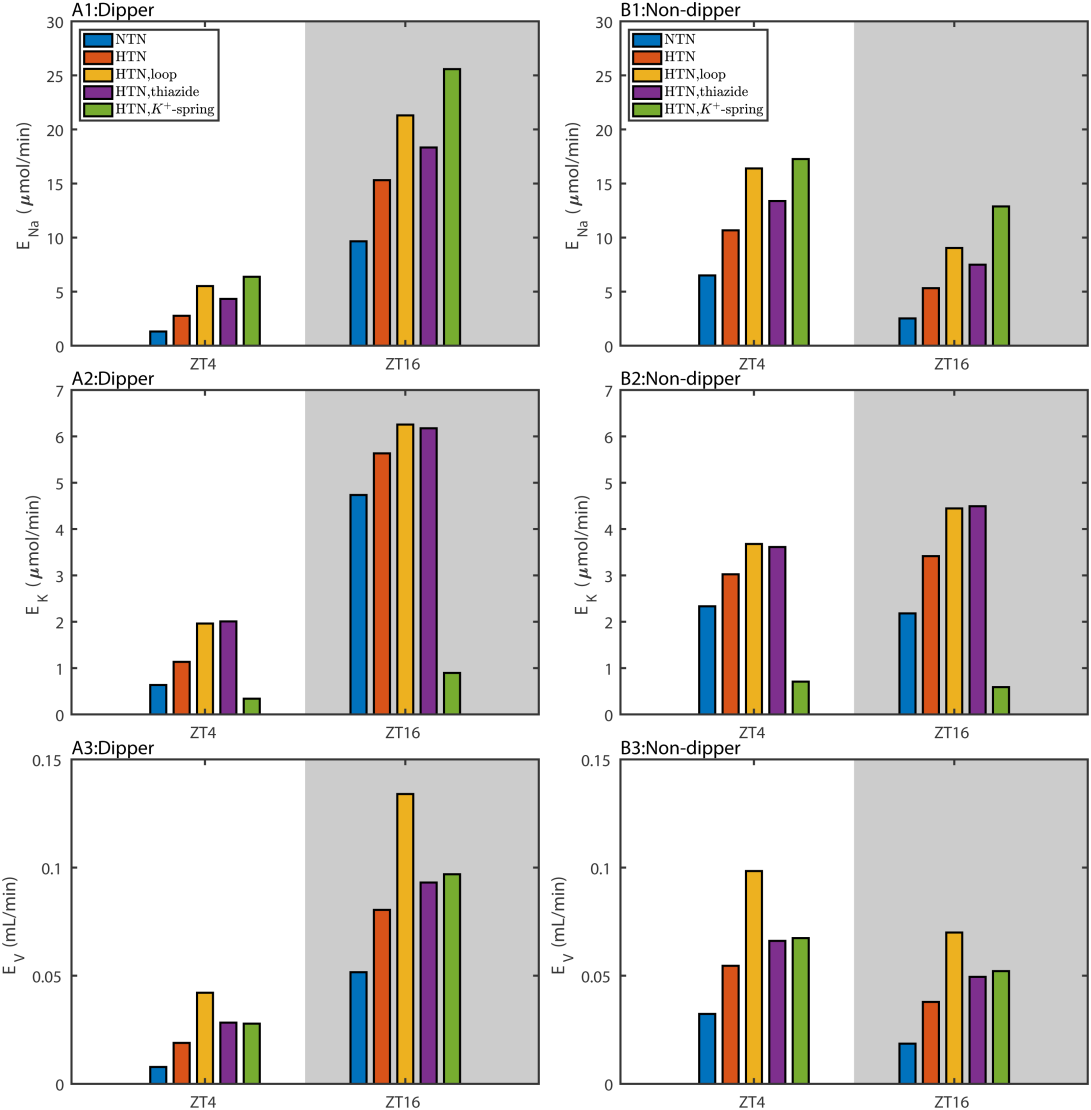
Predicted excretion rates at ZT4 (inactive phase) and ZT16 (active phase), obtained for the normotensive case (NTN), baseline hypertensive case (HTN), and hypertensive cases treated with a loop diuretic, a thiazide diuretic, and a K^+^-sparing diuretic. Left column **(A1—A3)**, SNGFR exhibits circadian rhythms (“Dipper”); right column **(B1—B3)**, constant SNGFR (“Non-dipper”). E_Na_, Na^+^ excretion; E_K_, K^+^ excretion; E_V_, urine output.

Model simulations are then conducted to investigate the effects of administration time of loop, thiazide, and K^+^-sparing diuretics on the kidney function of a rat with Ang II-induced hypertension. We consider separately dippers and non-dippers. Predicted Na^+^ excretion, K^+^ excretion, and urine output are shown for ZT4 and ZT16 in **Figure 8**. Each class of diuretics exerts the largest effect on Na^+^ reabsorption along the primary segment that expresses the transporter: thick ascending limb for loop diuretics, distal convoluted tubule for thiazide diuretics, and connecting tubule and collecting duct for K^+^-sparing diuretics. The lowered Na^+^ reabsorption enhances natriuresis throughout the day.

For dippers, Na^+^ excretion is larger at ZT16 (active (dark) phase) than ZT4 (inactive (light) phase) for all three diuretics (**Figure 8A1**). ZT16 is also when Na^+^ reabsorption is higher in each of these segments, but the influence of the larger SNGFR dominates. For non-dippers, where SNGFR stays constant throughout the day, Na^+^ excretion is larger at ZT4 (**Figure 8B1**), when the expression levels of the key Na^+^ transporters are lower. For both dippers and non-dippers, and for both ZT4 and ZT16, the natriuretic effect is the largest for the K^+^-sparing diuretics, because there are fewer segments downstream of its target site (connecting tubule and collecting duct) to compensate for the suppressed ENaC-mediated Na^+^ reabsorption.

Predicted urinary K^+^ excretion is higher at ZT16 than ZT4, with and without diuretics (**Figures 8A2**, **8B2**). For dippers, this is driven primarily by the higher SNGFR, as in the case of Na^+^ excretion. For non-dippers, this is due to the larger Na^+^ reabsorption, which drives K^+^ secretion. Loop and thiazide diuretics both enhance K^+^ excretion. K^+^-sparing diuretics essentially eliminate K^+^ secretion along the CNT, in fact switching it to a small amount of net reabsorption during the light phase (results not shown).

Water transport essentially follows Na^+^ transport along water-permeable segments. Thus, the predicted segmental water transport and urine output results are qualitatively similar to Na^+^, with the diuretic effect predicted to be larger at ZT16 for dippers but at ZT4 for non-dippers; see **Figures 8A3**, **8B3**. The diuretic effect is largest for loop diuretics, because it impairs the concentrating mechanism and lowers interstitial fluid osmolality, resulting in attenuated water reabsorption and elevated urine volume.

## 4 Discussion

Even though the occurrence of circadian rhythms is well known in several renal functions (Costello et al., 2022; Juffre and Gumz, 2024), their clinical importance has yet to be completely understood. In both humans and rodents, disruption of the circadian rhythms, including changes in time zone, is associated with an increased risk of cardiovascular disease (Douma and Gumz, 2018; Ramsey et al., 2023). Given the marked diurnal variations in urinary excretion, the plasma concentration or bioavailability of drugs that are eliminated by the kidneys might depend on the circadian expression of renal transporters, even if these drugs do not target renal transporters. The circadian rhythm is also known to affect the pathogenesis or progression of some renal diseases, such as renal tissue fibrosis (Chen et al., 2015) and kidney stones (Singh et al., 1990; Robert et al., 1994). The morbidity of patients with chronic kidney disease, as well as patients on hemodialysis or peritoneal dialysis, may be partially attributed to the disruption of the circadian rhythm of key renal functions (Koch et al., 2010; Russcher et al., 2015). The circadian rhythm is followed by blood pressure in humans as well, with values 10–15% lower during nighttime than during daytime, in a phenomenon known as dipping (Bankir et al., 2008). The absence of a nocturnal blood pressure decrease (i.e., non-dipping) is associated with target organ damage (Verdecchia et al., 1990). The interactions among the central and peripheral clocks, and the organ and tissue systems, are intriguing and complex. What are the underlying mechanisms that lead to the emergence of circadian rhythms in these processes? What are the clinical implications of the disappearance of these circadian rhythms? And how should pharmacological treatments be timed to maximize their effectiveness?

As a step toward answering these questions, we developed a detailed computational model of the circadian regulation of epithelial transport in a rat kidney. Because the present framework is mechanistic, it encodes directional relationships among physiological variables. However, these relationships are specified *a priori* based on current understanding and available data, and thus causal inferences drawn from the model are conditional on its structure and assumptions. Most published modeling studies of nephron epithelial transport focus on steady-state results (Layton et al., 2016; Hu et al., 2019; Hu and Layton, 2021; Stadt et al., 2023; Zheng and Layton, 2024), with a few exceptions such as Refs (Layton and Gumz, 2022; Dutta et al., 2023; Dutta and Layton, 2024; Stadt et al., 2024), where the circadian regulation of key renal transporters is represented. A key difference between those published studies (Layton and Gumz, 2022; Dutta et al., 2023) and the present model is that the former represent mouse kidneys, whereas the present model simulates a rat kidney. Also, the published studies (Layton and Gumz, 2022; Dutta et al., 2023; Dutta and Layton, 2024; Stadt et al., 2024) prescribe the circadian oscillations of the transporter activities, whereas in the present study, transporter activities are computed based on circadian protein levels, which are predicted by a detailed computational model of the renal circadian clock that simulates the interactions among Per, Cry, Bmal1, Clock, Ror, and Rev-Erb. By modeling the clock explicitly, we are able to predict the effect of clock gene knockdown on kidney function (see **Figure 1**).

By modeling the renal circadian clock and its regulation of transporter activities, our model can predict the extent to which Na^+^, K^+^ and water transport by a nephron is modulated by the renal circadian clock. A similar renal circadian clock was developed in Ref (Ogawa et al., 2011). to predict the circadian regulation of Na^+^ and water transport by a proximal convoluted tubule cell. That model (Ogawa et al., 2011) does not differentiate between homologs of Per and Cry. However, Per1 and Per2 have been shown to exert opposite effects on NHE3 expression (Saifur Rohman et al., 2005; Solocinski et al., 2015). Even though direct evidence is not currently available, it does not seem unreasonable to imagine that distinct roles of Per and Cry homologs on the circadian regulation of key renal transporters may be revealed in the future. Thus, the present model represents the Per and Cry homologs separately and allows the four protein complexes (PER1-CRY1, PER1-CRY2, PER2-CRY1, PER2-CRY2) to have different effects on transporter expression levels.

Emans et al. (2017) reported that wild-type rats exhibit a circadian rhythm in kidney tissue oxygenation, with lower levels during the light phase and higher levels during the dark phase, peaking at ZT16.9. Kidney oxygenation is a result of the balance of oxygen delivery and consumption, which in turn are determined by renal hemodynamics and metabolism, respectively. In contrast to the 24-hour circadian rhythm in wild-types, SNGFR and thus Na^+^ transport and excretion in Bmal1-knockout exhibit a double-peak, 12-hour rhythm (**Figure 5**). Given that renal metabolism is driven primarily by tubular active Na^+^ transport, we hypothesize that renal tissue oxygenation in Bmal1-knockout exhibits a different rhythm, with a 12-hour period instead of 24.

Our simulations of nephron function in a conditional Bmal1-knockdown highlight how disruption of the renal circadian clock can reshape both the *timing* and the *homeostatic strategy* of sodium handling. By suppressing Bmal1, the model reproduces a marked attenuation of circadian oscillations in multiple transporters together with the experimentally observed loss of rhythmic SNGFR. Importantly, the system does not respond uniformly across nephron segments: load-responsive transporters such as SGLT1, NKCC2, and NCC exhibit elevated mean expression, consistent with a compensatory increase in reabsorptive capacity when filtered load becomes chronically mis-timed and elevated during the rest phase; see **Figure 4**. In this framework, the kidney adapts less through coordinated rhythmic trafficking and more through tonic upregulation of key transport pathways that buffer persistent delivery signals. In contrast, the relative preservation (or eventual reduction under complete knockout) of NHE3 and ENaC underscores the presence of distinct regulatory constraints, with proximal transport remaining tightly coupled to flow and luminal chemistry, and distal ENaC subject to stringent aldosterone–K^+^ balance control to prevent over-retention when upstream reabsorption increases.

Functionally, these differential adaptations translate into a profound alteration of Na^+^ excretion dynamics. The emergence of two daily peaks in urinary Na^+^ excretion (**Figure 5**) and the exaggerated inactive-phase natriuresis reflect the combined effects of ultradian SNGFR behavior and segment-specific transporter remodeling. The predicted blunting—or reversal—of the normal “dipping” pattern provides a mechanistic link between circadian clock disruption and impaired temporal partitioning of Na^+^ balance, consistent with experimental observations in Bmal1-deficient animals. Clinically, such misalignment may be relevant to salt-sensitive hypertension and non-dipping blood pressure phenotypes, where inappropriate sodium retention or excretion timing is increasingly recognized as pathogenic. More broadly, these results suggest that renal clock genes influence not only the amplitude of transporter rhythms but also the set-point of tubular transport capacity, with implications for chronotherapy and for understanding how circadian disruption (e.g., shift work, sleep disorders, aging) predisposes to cardiometabolic disease through altered renal Na^+^ handling.

The present framework isolates renal epithelial transport and selected Ang II–mediated regulatory effects, rather than attempting to represent the full neurohormonal control of Na^+^ balance.

By incorporating circadian rhythms into our previously developed hypertensive rat kidney transport framework (Zheng and Layton, 2024), the present model provides new mechanistic insight into how hypertension reshapes not only the magnitude but also the timing of renal Na^+^, K^+^, and water handling. Importantly, the model further identifies oscillations in SNGFR as a key determinant of the phase and amplitude of daily excretory rhythms in both normotensive and hypertensive states. These simulations underscore the clinical relevance of circadian regulation as a determinant of renal sodium handling in hypertension. A growing body of evidence suggests that hypertensive disease is frequently accompanied not only by increased distal Na^+^ reabsorption, but also by impaired day–night variation in renal hemodynamics and excretory function (Burnier et al., 2007; Nguyen et al., 2013; Liu et al., 2023). Our results support the idea that disruption of SNGFR rhythmicity may represent a mechanistic link between hypertension and the loss of normal temporal partitioning of natriuresis. In particular, a kidney that no longer exhibits appropriate circadian variation in filtration may rely disproportionately on tubular transporter rhythms to maintain sodium balance, with the consequence that Na^+^ excretion becomes phase-shifted and less effectively aligned with behavioral cycles. Such temporal misalignment is likely to contribute to clinically recognized non-dipping phenotypes, in which nocturnal sodium retention and altered pressure–natriuresis relationships are associated with heightened cardiovascular risk (Fujii et al., 1999; Huart et al., 2023).

From a therapeutic perspective, these findings emphasize that the efficacy of diuretics may depend not only on drug class and nephron target, but also on circadian phenotype. Chronotherapy trials in hypertension have suggested that dosing time can influence blood pressure control, yet the mechanistic basis has remained incompletely understood. The present model provides a physiological explanation: when SNGFR retains circadian oscillations (“dippers”), diuretic responsiveness is greatest during periods of elevated filtered load, whereas in “non-dippers,” optimal natriuresis may instead occur when transporter expression is lowest and compensatory capacity is reduced. Thus, non-dipping may alter the timing of maximal physiological diuretic responsiveness, implying that uniform morning or evening dosing strategies may fail to account for substantial inter-individual variation in renal circadian function. More broadly, these results support the concept that circadian diagnostics, such as dipping status or biomarkers of renal clock disruption, could inform personalized antihypertensive therapy.

The model further highlights clinically important distinctions among diuretic classes in the setting of circadian and hypertensive remodeling. K^+^-sparing agents are predicted to exert particularly strong natriuretic effects because inhibition at the level of ENaC leaves minimal downstream capacity for compensation, while simultaneously mitigating kaliuresis, an important consideration in patients at risk for diuretic-induced hypokalemia. Conversely, loop diuretics exert the greatest impact on urine volume through disruption of the medullary concentrating mechanism, consistent with their established clinical potency but also their propensity for volume depletion. Taken together, these findings suggest that circadian control of filtration and transporter abundance is not merely a physiological curiosity, but a clinically relevant modifier of diuretic response, electrolyte balance, and ultimately blood pressure regulation. Incorporating renal circadian biology into treatment paradigms may improve management of salt-sensitive and non-dipping hypertension, particularly in patients with disrupted sleep–wake cycles, aging-associated clock dysfunction, or resistant disease.

### 4.1 Limitations and future extensions

The effectiveness of antihypertensive therapy is ultimately judged by its capacity to lower arterial blood pressure. Accordingly, the present framework should be interpreted as a mechanistic nephron-scale model designed to isolate selected circadian and transport-mediated mechanisms, rather than as a comprehensive representation of whole-body cardiovascular and endocrine regulation. The nephron model used in the present study provides detailed predictions of segmental transport, urinary excretion, and urine output, but it does not explicitly represent extracellular fluid volume or systemic blood pressure. In particular, the model does not incorporate several key regulatory pathways known to contribute to blood pressure control, including the renin–angiotensin–aldosterone system, renal sympathetic nerve activity, oxidative stress and nitric oxide signaling, and the endothelin system. Many of these mechanisms act, at least in part, through the kidney, and their interactions with tubular transport may substantially modulate the net hemodynamic response to diuretic therapy.

Although Ang II–dependent changes in transporter activity are incorporated, the model does not explicitly represent time-varying hormonal signals. In particular, circadian rhythms in circulating hormones such as renin and aldosterone are not modeled directly, despite their known roles in regulating renal transporter activity and sodium balance. Instead, their downstream effects are represented implicitly through prescribed or fitted changes in transporter activity. This approach enables the isolation of tubular and filtration-driven mechanisms but does not capture potential feedback between hormonal rhythms and renal function, which may contribute to the circadian regulation of sodium handling *in vivo*.

In addition, the simulations of diuretic action assume an immediate attainment of steady-state inhibition of target transporters, without explicitly modeling drug pharmacokinetics or pharmacodynamics. In reality, drug absorption, distribution, metabolism, and excretion introduce time delays and variability in drug concentration, which may influence both the timing and magnitude of the natriuretic response. Incorporating pharmacokinetic–pharmacodynamic coupling would provide a more realistic representation of drug effects and may alter the predicted optimal timing of administration. Furthermore, the model assumes that circadian temporal patterns of transporter regulation are preserved under hypertensive conditions, due to the limited availability of time-resolved experimental data. While this assumption enables isolation of baseline remodeling effects, it may overlook potential hypertension-induced alterations in circadian phase or amplitude. Model predictions were found to be qualitatively robust to moderate variations in circadian timing, but incorporating such effects remains an important direction for future work.

More broadly, while the model enables *in silico* perturbations that isolate the effects of individual mechanisms, such as circadian variation in SNGFR or transporter expression, these interventions reflect causal relationships within the model rather than direct experimental evidence. As such, the identification of SNGFR oscillations as a dominant driver of excretory rhythms should be interpreted as a mechanistic hypothesis that warrants further experimental validation.

Finally, the translation of these findings to clinical practice requires caution. Although the model suggests that the optimal timing of diuretic administration may depend on circadian phenotype (e.g., dipping status), real-world treatment decisions are additionally shaped by patient adherence, convenience, sleep disruption, work schedules, tolerability, and other patient-specific considerations. Thus, while the present results provide a physiological rationale for chronotherapy, their clinical implementation would require integration with practical and patient-centered factors, as well as validation in experimental and clinical studies.

A natural extension of the present work is therefore to embed these nephron-scale circadian transport models within whole-body frameworks of blood pressure regulation (Ahmed et al., 2019; Leete and Layton, 2019). Such integrative models would account for systemic feedback control and provide a mechanistically grounded link between time-of-day variation in renal excretory responses, extracellular fluid volume, and blood pressure outcomes. In addition, extending the model to incorporate sex differences represents an important future direction. The present model simulates kidney function in a male rat; future studies should extend this framework to female physiology, followed by sex-specific whole-body models of circadian blood pressure regulation. Such extensions may yield further insight into the mechanisms underlying sex differences in hypertension and its treatment.

## Data Availability

The original contributions presented in the study are included in the article/**Supplementary Material**. Further inquiries can be directed to the corresponding author.
