# Supplementary material for: Circadian rhythms in glomerular filtration govern natriuresis and diuretic responsiveness
Source: Front Physiol. 2026 Jun 2;17:1828410. doi: 10.3389/fphys.2026.1828410 (PMC13268944; doi:10.3389/fphys.2026.1828410)
Supplement: Supplementary file 1 [file DataSheet1.pdf]

Appendix A

A1 Governing equations overview

Eq. A1-A21 are 25 governing equations of 12 mRNAs: Per1, Per2, Cry1, Cry2, Rev-Erb, Ror, Bmal1, NHE3, NKCC2, SGLT1,NCC, and ENaC; 7 proteins: PER1, PER2, CRY1, CRY2, REV-ERB, ROR, and BMAL1; and 5 protein complexes: PER1-CRY1, PER2-CRY1, PER1-CRY2, PER2-CRY2, and CLOCK-BMAL1. Because in our formulation all mRNA are regualted by 5 protein complexes in an analogous pattern, we unify the formulation by defining a regulation factor $\mathrm{F_X}$ (Eq. A13) where X is the name of mRNA. All parameters are listed with description in Section A4.

A2 mRNA equations

$$\frac{d[\mathrm{Per1}]}{dt} = -\mathrm{dm\_Per1}\cdot[\mathrm{Per1}] + \mathrm{V\_Per1}\cdot \mathrm{F_{Per1}} \tag{A1}$$

$$\frac{d[\mathrm{Per2}]}{dt} = -\mathrm{dm\_Per2}\cdot[\mathrm{Per2}] + \mathrm{V\_Per2}\cdot \mathrm{F_{Per2}} \tag{A2}$$

$$\frac{d[\mathrm{Cry1}]}{dt} = -\mathrm{dm\_Cry1}\cdot[\mathrm{Cry1}] + \frac{\mathrm{V\_Cry1}\cdot \mathrm{F_{Cry1}}}{(1+(\frac{\mathrm{REV\_ERB}}{\mathrm{Ki\_Cry1\_rev}})^{\mathrm{hill\_Cry1\_REV}})} \tag{A3}$$

$$\frac{d[\mathrm{Cry2}]}{dt} = -\mathrm{dm\_Cry2}\cdot[\mathrm{Cry2}] + \frac{\mathrm{V\_Cry2}\cdot \mathrm{F_{Cry2}}}{(1+(\frac{\mathrm{REV\_ERB}}{\mathrm{Ki\_Cry2\_rev}})^{\mathrm{hill\_Cry2\_REV}})} \tag{A4}$$

$$\frac{d[\mathrm{Rev\text{-}Erb}]}{dt} = -\mathrm{dm\_rev}\cdot[\mathrm{Rev\text{-}Erb}] + \mathrm{V\_Rev}\cdot \mathrm{F_{rev}} \tag{A5}$$

$$\frac{d[\mathrm{Ror}]}{dt} = -\mathrm{dm\_Ror}\cdot[\mathrm{Ror}] + \mathrm{V\_Ror}\cdot \mathrm{F_{Ror}} \tag{A6}$$

$$\frac{d[\mathrm{Bmal1}]}{dt} = -\mathrm{dm\_Bmal1}\cdot[\mathrm{Bmal1}] + \frac{\mathrm{V\_Bmal1}\cdot(1+\mathrm{fold\_Bmal1}\cdot(\frac{[\mathrm{ROR}]}{\mathrm{Ka\_Bmal1\_Ror}})^{\mathrm{hill\_Bmal1\_ROR}})}{1+(\frac{[\mathrm{REV\text{-}ERB}]}{\mathrm{Ki\_Bmal1\_rev}})^{\mathrm{hill\_Bmal1\_REV}}+(\frac{[\mathrm{ROR}]}{\mathrm{Ka\_Bmal1\_Ror}})^{\mathrm{hill\_Bmal1\_ROR}}} \tag{A7}$$

$$\begin{aligned}\frac{d[\mathrm{NHE3}]}{dt} &= -\mathrm{dm\_NHE3}\cdot[\mathrm{NHE3}] + \mathrm{V\_NHE3}\cdot(1+\mathrm{fold\_NHE3}\cdot(\frac{[\mathrm{CLOCK-BMAL1}]}{\mathrm{Ka\_NHE3\_cb}})^{\mathrm{hill\_NHE3\_cb}}\cdot(1\\ &+(\frac{[\mathrm{PER1-CRY2}]}{\mathrm{Ka\_NHE3\_P1C2}})^{\mathrm{hill\_NHE3\_p1c2}}))/(1+(\frac{[\mathrm{CLOCK-BMAL1}]}{\mathrm{Ka\_NHE3\_cb}})^{\mathrm{hill\_NHE3\_cb}}\cdot(1+(\frac{[\mathrm{PER1-CRY2}]}{\mathrm{Ka\_NHE3\_P1C2}})^{\mathrm{hill\_NHE3\_p1c2}})\\ &\cdot(1+(\frac{[\mathrm{PER2\_CRY1}]}{\mathrm{Ki\_NHE3\_p2c1}})^{\mathrm{hill\_NHE3\_p2c1}})\cdot(1+(\frac{[\mathrm{PER2\_CRY2}]}{\mathrm{Ki\_NHE3\_p2c2}})^{\mathrm{hill\_NHE3\_p2c2}})\cdot(1+\frac{[\mathrm{PER1-CRY1}]}{\mathrm{Ki\_NHE3\_p1c1}}+)^{\mathrm{hill\_NHE3\_p1c1}})\end{aligned} \tag{A8}$$

$$\frac{d[\mathrm{NKCC2}]}{dt} = -\mathrm{dm\_NKCC2}\cdot[\mathrm{NKCC2}] + \mathrm{V\_NKCC2}\cdot \mathrm{F_{NKCC2}} \tag{A9}$$

$$\begin{aligned}\frac{d[\mathrm{SGLT1}]}{dt} =& -\mathrm{dm\_SGLT1}\cdot[\mathrm{SGLT1}]\\ &+\frac{\mathrm{V\_SGLT1}(1+\mathrm{fold\_SGLT1}\cdot(\frac{[\mathrm{PER1-CRY1}]}{\mathrm{Ka\_SGLT1\_p1c1}}+\frac{[\mathrm{PER1-CRY2}]}{\mathrm{Ka\_SGLT1\_p1c2}})^{\mathrm{hill\_NCC\_p1c}})}{1+(\frac{[\mathrm{CLOCK-BMAL1}]}{\mathrm{Ki\_SGLT1\_cb}})^{\mathrm{hill\_SGLT1\_cb}}+(\frac{[\mathrm{PER1-CRY1}]}{\mathrm{Ka\_SGLT1\_p1c1}}+\frac{[\mathrm{PER1-CRY2}]}{\mathrm{Ka\_SGLT1\_p1c2}})^{\mathrm{hill\_NCC\_p1c}}}\end{aligned} \tag{A10}$$

$$\begin{aligned}\frac{d[\mathrm{NCC}]}{dt} =& -\mathrm{dm\_NCC}\cdot[\mathrm{NCC}]\\ &+\frac{\mathrm{V\_NCC}(1+\mathrm{fold\_NCC}\cdot(\frac{[\mathrm{PER1-CRY1}]}{\mathrm{Ka\_NCC\_p1c1}}+\frac{[\mathrm{PER1-CRY2}]}{\mathrm{Ka\_NCC\_p1c2}})^{\mathrm{hill\_NCC\_p1c}})}{1+(\frac{[\mathrm{CLOCK-BMAL1}]}{\mathrm{Ki\_NCC\_cb}})^{\mathrm{hill\_NCC\_cb}}+(\frac{[\mathrm{PER1-CRY1}]}{\mathrm{Ka\_NCC\_p1c1}}+\frac{[\mathrm{PER1-CRY2}]}{\mathrm{Ka\_NCC\_p1c2}})^{\mathrm{hill\_NCC\_p1c}}}\end{aligned} \tag{A11}$$

$$\frac{d[\mathrm{ENaC}]}{dt} = -\mathrm{dm\_ENaC}\cdot[\mathrm{ENaC}]$$

$$
+\frac{\mathrm{V\_ENaC}(1+\mathrm{fold\_ENaC}\cdot(\frac{[\mathrm{CLOCK-BMAL1}]}{\mathrm{Ka\_ENaC\_cb}})^{\mathrm{hill\_ENaC\_cb}}(1+(\frac{[\mathrm{PER1-CRY1}]}{\mathrm{Ka\_ENaC\_p1c1}}+\frac{[\mathrm{PER1-CRY2}]}{\mathrm{Ka\_ENaC\_p1c2}})^{\mathrm{hill\_ENaC\_p1c}})}{1+(\frac{[\mathrm{CLOCK-BMAL1}]}{\mathrm{Ka\_ENaC\_cb}})^{\mathrm{hill\_ENaC\_cb}}(1+(\frac{[\mathrm{PER1-CRY1}]}{\mathrm{Ka\_ENaC\_p1c1}}+\frac{[\mathrm{PER1-CRY2}]}{\mathrm{Ka\_ENaC\_p1c2}})^{\mathrm{hill\_ENaC\_p1c}})} \tag{A12}
$$

$$
\begin{aligned}
\mathrm{F_X} = (1+\mathrm{fold\_X}\cdot(\frac{[\mathrm{CLOCK\_BMAL1}]}{\mathrm{Ka\_X\_cb}})^{\mathrm{hill\_X\_cb}}))/(1+(\frac{[\mathrm{CLOCK\_BMAL1}]}{\mathrm{Ka\_X\_cb}})^{\mathrm{hill\_X\_cb}} \\
\cdot(1+(\frac{[\mathrm{PER1\_CRY1}]}{\mathrm{Ki\_X\_p1c1}})^{\mathrm{hill\_X\_p1c1}})\cdot(1+(\frac{[\mathrm{PER1\_CRY2}]}{\mathrm{Ki\_X\_p1c2}})^{\mathrm{hill\_X\_p1c2}})\cdot \\
(1+(\frac{[\mathrm{PER2\_CRY1}]}{\mathrm{Ki\_X\_p2c1}})^{\mathrm{hill\_X\_p2c1}})\cdot(1+(\frac{[\mathrm{PER2\_CRY2}]}{\mathrm{Ki\_X\_p2c2}})^{\mathrm{hill\_X\_p2c2}}))
\end{aligned} \tag{A13}
$$

A3 Protein equations

$$
\begin{aligned}
\frac{\mathrm{d[PER1]}}{\mathrm{dt}} &= -\mathrm{dp\_PER1}\cdot[\mathrm{PER1}]+\mathrm{kp\_PER1}\cdot[\mathrm{Per1}]-\mathrm{kass\_p1c1}\cdot[\mathrm{PER1}]\cdot[\mathrm{CRY1}] \\
&-\mathrm{kass\_p1c2}\cdot[\mathrm{PER1}]\cdot[\mathrm{CRY2}]+\mathrm{kdiss\_p1c1}\cdot[\mathrm{PER1\text{-}CRY1}]+\mathrm{kdiss\_p1c2}\cdot[\mathrm{PER1\text{-}CRY2}]
\end{aligned} \tag{A14}
$$

$$
\begin{aligned}
\frac{\mathrm{d[PER2]}}{\mathrm{dt}} &= -\mathrm{dp\_PER2}\cdot[\mathrm{PER2}]+\mathrm{kp\_PER2}\cdot[\mathrm{Per2}]-\mathrm{kass\_p2c1}\cdot[\mathrm{PER2}]\cdot[\mathrm{CRY1}] \\
&-\mathrm{kass\_p2c2}\cdot[\mathrm{PER2}]\cdot[\mathrm{CRY2}]+\mathrm{kdiss\_p2c1}\cdot[\mathrm{PER2\text{-}CRY1}]+\mathrm{kdiss\_p2c2}\cdot[\mathrm{PER2\text{-}CRY2}]
\end{aligned} \tag{A15}
$$

$$
\begin{aligned}
\frac{\mathrm{d[CRY1]}}{\mathrm{dt}} &= -\mathrm{dp\_CRY1}\cdot[\mathrm{CRY1}]+\mathrm{kp\_CRY1}\cdot[\mathrm{Cry1}]-\mathrm{kass\_p1c1}\cdot[\mathrm{PER1}]\cdot[\mathrm{CRY1}] \\
&-\mathrm{kass\_p2c1}\cdot[\mathrm{PER2}]\cdot[\mathrm{CRY1}]+\mathrm{kdiss\_p1c1}\cdot[\mathrm{PER1\text{-}CRY1}]+\mathrm{kdiss\_p2c1}\cdot[\mathrm{PER2\text{-}CRY1}]
\end{aligned} \tag{A16}
$$

$$
\begin{aligned}
\frac{\mathrm{d[CRY2]}}{\mathrm{dt}} &= -\mathrm{dp\_CRY2}\cdot[\mathrm{CRY2}]+\mathrm{kp\_CRY2}\cdot[\mathrm{Cry2}]-\mathrm{kass\_p1c2}\cdot[\mathrm{PER1}]\cdot[\mathrm{CRY2}] \\
&-\mathrm{kass\_p2c2}\cdot[\mathrm{PER2}]\cdot[\mathrm{CRY2}]+\mathrm{kdiss\_p1c2}\cdot[\mathrm{PER1\text{-}CRY2}]+\mathrm{kdiss\_p2c2}\cdot[\mathrm{PER2\text{-}CRY2}]
\end{aligned} \tag{A17}
$$

$$
\frac{\mathrm{d[PER1\text{-}CRY1]}}{\mathrm{dt}} = \mathrm{kass\_p1c1}\cdot[\mathrm{PER1}]\cdot[\mathrm{CRY1}]-\mathrm{kdiss\_p1c1}\cdot[\mathrm{PER1\text{-}CRY1}]-\mathrm{d\_p1c1}\cdot[\mathrm{PER1\text{-}CRY1}] \tag{A18}
$$

$$
\frac{\mathrm{d[PER2\text{-}CRY1]}}{\mathrm{dt}} = \mathrm{kass\_p2c1}\cdot[\mathrm{PER2}]\cdot[\mathrm{CRY1}]-\mathrm{kdiss\_p2c1}\cdot[\mathrm{PER2\text{-}CRY1}]-\mathrm{d\_p2c1}\cdot[\mathrm{PER2\text{-}CRY1}] \tag{A19}
$$

$$
\frac{\mathrm{d[PER1\text{-}CRY2]}}{\mathrm{dt}} = \mathrm{kass\_p1c2}\cdot[\mathrm{PER1}]\cdot[\mathrm{CRY2}]-\mathrm{kdiss\_p1c2}\cdot[\mathrm{PER1\text{-}CRY2}]-\mathrm{d\_p1c2}\cdot[\mathrm{PER1\text{-}CRY2}] \tag{A20}
$$

$$
\frac{\mathrm{d[PER2\text{-}CRY2]}}{\mathrm{dt}} = \mathrm{kass\_p2c2}\cdot[\mathrm{PER2}]\cdot[\mathrm{CRY2}]-\mathrm{kdiss\_p2c2}\cdot[\mathrm{PER2\text{-}CRY2}]-\mathrm{d\_p2c2}\cdot[\mathrm{PER2\text{-}CRY2}] \tag{A21}
$$

$$
\frac{\mathrm{d[REV\text{-}ERB]}}{\mathrm{dt}} = -\mathrm{dp\_REV}\cdot[\mathrm{REV\text{-}ERB}]+\mathrm{kp\_REV}\cdot[\mathrm{Rev\text{-}Erb}] \tag{A22}
$$

$$\frac{\mathrm{d[ROR]}}{\mathrm{dt}} = -\mathrm{dp\_ROR} \cdot [\mathrm{ROR}] + \mathrm{kp\_ROR} \cdot [\mathrm{Ror}] \tag{A23}$$

$$\frac{\mathrm{d[BMAL1]}}{\mathrm{dt}} = -\mathrm{dp\_BMAL1} \cdot [\mathrm{BMAL1}] + \mathrm{kp\_BMAL1} \cdot [\mathrm{Bmal1}] - \mathrm{kass\_cb} \cdot [\mathrm{BMAL1}] + \mathrm{kdiss\_cb} \cdot [\mathrm{CLOCK\text{-}BMAL1}] \tag{A24}$$

$$\frac{\mathrm{d[CLOCK\text{-}BMAL1]}}{\mathrm{dt}} = -\mathrm{d\_cb} \cdot [\mathrm{CLOCK\text{-}BMAL1}] + \mathrm{kass\_cb} \cdot [\mathrm{BMAL1}] - \mathrm{kdiss\_cb} \cdot [\mathrm{CLOCK\text{-}BMAL1}] \tag{A25}$$

A4 Baseline parameters

Table A1: Degradation rates (in hour^{-1}) of protein complexes, mRNAs, and proteins .

**Parameter**	**Value**	**Description**
d_cb	0.098775151	CLOCK-BMAL1 complex degradation rate
d_p1c1	2.747914868	PER1-CRY1 protein complex degradation rate
d_p1c2	3.857884688	PER1-CRY2 protein complex degradation rate
d_p2c1	1.98150769	PER2-CRY1 protein complex degradation rate
d_p2c2	0.009962143	PER2-CRY2 protein complex degradation rate
dm_Bmal1	10.29999143	Bmal1 mRNA degradation rate
dm_Cry1	1.680357077	Cry1 mRNA degradation rate
dm_Cry2	1.102297307	Cry2 mRNA degradation rate
dm_Per1	0.393284589	Per1 mRNA degradation rate
dm_Per2	0.199538876	Per2 mRNA degradation rate
dm_Rev	0.202152455	Rev mRNA degradation rate
dm_Ror	0.733726737	Ror mRNA degradation rate
dm_NHE3	2.6212974828	NHE3 mRNA degradation rate
dm_NKCC2	0.147706095	NKCC2 mRNA degradation rate
dm_SGLT1	0.9719928901	SGLT1 mRNA degradation rate
dm_NCC	0.11428499820	NCC mRNA degradation rate
dm_ENaC	1.4666546516	ENaC mRNA degradation rate
dp_BMAL1	0.1223564	BMAL1 protein degradation rate
dp_CRY1	0.07170199	CRY1 protein degradation rate
dp_CRY2	0.063886143	CRY2 protein degradation rate
dp_PER1	2.118148503	PER1 protein degradation rate
dp_PER2	1.092137167	PER2 protein degradation rate
dp_REV	1.54249893	REV-ERB protein degradation rate
dp_ROR	0.110251246	ROR protein degradation rate

Table A2: Activation ratios (dimensionless).

Parameter	Value	Description
fold_Bmal1	29.23079974	Activation ratio of Bmal1 by ROR
fold_Cry1	17.45987491	Activation ratio of Cry1 by CLOCK-BMAL1
fold_Cry2	13.21473162	Activation ratio of Cry2 by CLOCK-BMAL1
fold_Per1	2.431717595	Activation ratio of Per1 by CLOCK-BMAL1
fold_Per2	3.308354134	Activation ratio of Per2 by CLOCK-BMAL1
fold_Rev	118.5947203	Activation ratio of Rev by CLOCK-BMAL1
fold_Ror	5.603448203	Activation ratio of Ror by CLOCK-BMAL1
fold_NHE3	5.63209402975596	Activation ratio of NHE3 by CLOCK-BMAL1 and PER1-CRY2
fold_NKCC2	0.385311524	Activation ratio of NKCC2 by CLOCK-BMAL1
fold_SGLT1	2.36457412221910	Activation ratio of SGLT1 by CLOCK-BMAL1
fold_NCC	1.026401864136	Activation ratio of NCC by PER1-CRY1 and PER1-CRY2
fold_ENaC	6.2735611435	Activation ratio of ENaC by CLOCK-BMAL1

Table A3: Hill coefficients (dimensionless).

Parameter	Value	Description
hill_Bmal1_REV	6.315290784	Hill coefficient regulation of Bmal1 by REV-ERB
hill_Bmal1_ROR	10.19219221	Hill coefficient regulation of Bmal1 by ROR
hill_Cry1_cb	4.565210968	Hill coefficient regulation of Cry1 by CLOCK-BMAL1
hill_Cry1_p1c1	1.768766266	Hill coefficient regulation of Cry1 by PER1-CRY1
hill_Cry1_p1c2	1.438000996	Hill coefficient regulation of Cry1 by PER1-CRY2
hill_Cry1_p2c1	1.589908884	Hill coefficient regulation of Cry1 by PER2-CRY1
hill_Cry1_p2c2	1.830362004	Hill coefficient regulation of Cry1 by PER2-CRY2
hill_Cry1_Rev	4.820086463	Hill coefficient regulation of Cry1 by REV-ERB
hill_Cry2_cb	1.367823416	Hill coefficient regulation of Cry2 by CLOCK-BMAL1
hill_Cry2_p1c1	2.1098479	Hill coefficient regulation of Cry2 by PER1-CRY1
hill_Cry2_p1c2	2.026033401	Hill coefficient regulation of Cry2 by PER1-CRY2
hill_Cry2_p2c1	1.602187775	Hill coefficient regulation of Cry2 by PER2-CRY1
hill_Cry2_p2c2	1.655287085	Hill coefficient regulation of Cry2 by PER2-CRY2
hill_Cry2_rev	1.032736435	Hill coefficient regulation of Cry2 by REV-ERB
hill_Per1_cb	4.096754782	Hill coefficient regulation of Per1 by CLOCK-BMAL1
hill_Per1_p1c1	4.137208965	Hill coefficient regulation of Per1 by PER1-CRY1
hill_Per1_p1c2	4.352895626	Hill coefficient regulation of Per1 by PER1-CRY2
hill_Per1_p2c1	4.556232531	Hill coefficient regulation of Per1 by PER2-CRY1
hill_Per1_p2c2	3.006414519	Hill coefficient regulation of Per1 by PER2-CRY2
hill_Per2_cb	5.973526978	Hill coefficient regulation of Per2 by CLOCK-BMAL1
hill_Per2_p1c1	4.917946201	Hill coefficient regulation of Per2 by PER1-CRY1
hill_Per2_p1c2	4.094774755	Hill coefficient regulation of Per2 by PER1-CRY2
hill_Per2_p2c1	4.287994314	Hill coefficient regulation of Per2 by PER2-CRY1
hill_Per2_p2c2	5.627510726	Hill coefficient regulation of Per2 by PER2-CRY2
hill_Rev_cb	13.59046465	Hill coefficient regulation of Rev-Erb by CLOCK-BMAL1
hill_Rev_p1c1	4.593824878	Hill coefficient regulation of Rev-Erb by PER1-CRY1
hill_Rev_p1c2	4.473939935	Hill coefficient regulation of Rev-Erb by PER1-CRY2
hill_Rev_p2c1	4.502871061	Hill coefficient regulation of Rev-Erb by PER2-CRY1
hill_Rev_p2c2	4.474437607	Hill coefficient regulation of Rev-Erb by PER2-CRY2
hill_Ror_cb	11.23225359	Hill coefficient regulation of Ror by CLOCK-BMAL1
hill_Ror_p1c1	3.346138809	Hill coefficient regulation of Ror by PER1-CRY1
hill_Ror_p1c2	4.279505041	Hill coefficient regulation of Ror by PER1-CRY2
hill_Ror_p2c1	3.213895403	Hill coefficient regulation of Ror by PER2-CRY1
hill_Ror_p2c2	4.047251298	Hill coefficient regulation of Ror by PER2-CRY2
hill_NHE3_cb	2.2098682284	Hill coefficient regulation of NHE3 by CLOCK-BMAL1
hill_NHE3_p1c1	4.0457153929	Hill coefficient regulation of NHE3 by PER1-CRY1
hill_NHE3_p1c2	5.8872697770	Hill coefficient regulation of NHE3 by PER1-CRY2
hill_NHE3_p2c1	5.1885742129	Hill coefficient regulation of NHE3 by PER2-CRY1
hill_NHE3_p2c2	1.7481499102	Hill coefficient regulation of NHE3 by PER2-CRY2
hill_NKCC2_cb	21.63335325	Hill coefficient regulation of NKCC2 by CLOCK-BMAL1
hill_NKCC2_p1c1	10.57552098	Hill coefficient regulation of NKCC2 by PER1-CRY1
hill_NKCC2_p1c2	11.3012353	Hill coefficient regulation of NKCC2 by PER1-CRY2
hill_NKCC2_p2c1	11.0258738	Hill coefficient regulation of NKCC2 by PER2-CRY1
hill_NKCC2_p2c2	15.74026414	Hill coefficient regulation of NKCC2 by PER2-CRY2
hill_SGLT1_cb	1.8701185743	Hill coefficient regulation of SGLT1 by CLOCK-BMAL1
hill_SGLT1_p1c	5.86981584935250	Hill coefficient regulation of SGLT1 by PER1-CRY1 and PER1-CRY2
hill_NCC_cb	2.1869392091	Hill coefficient regulation of NCC by CLOCK-BMAL1
hill_NCC_p1c	4.8511288196	Hill coefficient regulation of NCC by PER1-CRY1 and PER1-CRY2
hill_ENaC_cb	6.4999347889	Hill coefficient regulation of ENaC by CLOCK-BMAL1
hill_ENaC_p1c	10.4928793515923	Hill coefficient regulation of ENaC by PER1-CRY1 and PER1-CRY2

Table A4: Regulation thresholds (dimensionless).

Parameter	Value	Description
Ka_Bmal1_ror	0.788134198	Regulation threshold of Bmal1 by ROR
Ka_Cry1_cb	2.965895772	Regulation threshold of Cry1 by CLOCK-BMAL1
Ka_Cry2_cb	9.818058078	Regulation threshold of Cry2 by CLOCK-BMAL1
Ka_Per1_cb	3.045974034	Regulation threshold of Per1 by CLOCK-BMAL1
Ka_Per2_cb	0.996030215	Regulation threshold of Per2 by CLOCK-BMAL1
Ka_Rev_cb	2.407381652	Regulation threshold of Rev-Erb by CLOCK-BMAL1
Ka_Ror_cb	2.531369156	Regulation threshold of Ror by CLOCK-BMAL1
Ka_NHE3_cb	3.0427238397	Regulation threshold of NHE3 by CLOCK-BMAL1
Ka_NKCC2_cb	1.238599283	Regulation threshold of NKCC2 by CLOCK-BMAL1
Ki_SGLT1_cb	5.414004356	Regulation threshold of SGLT1 by CLOCK-BMAL1
Ki_NCC_cb	0.5108645898	Regulation threshold of NCC by CLOCK-BMAL1
Ka_ENaC_cb	3.2433974568	Regulation threshold of ENaC by CLOCK-BMAL1
Ki_Bmal1_rev	0.000960248	Regulation threshold of Bmal1 by REV-ERB
Ki_Cry1_p1c1	2.559548048	Regulation threshold of Cry1 by PER1-CRY1
Ki_Cry1_p1c2	2.536630442	Regulation threshold of Cry1 by PER1-CRY2
Ki_Cry1_p2c1	2.455448644	Regulation threshold of Cry1 by PER2-CRY1
Ki_Cry1_p2c2	3.054560489	Regulation threshold of Cry1 by PER2-CRY2
Ki_Cry1_rev	3.597110237	Regulation threshold of Cry1 by REV-ERB
Ki_Cry2_p1c1	2.852145929	Regulation threshold of Cry2 by PER1-CRY1
Ki_Cry2_p1c2	3.094816119	Regulation threshold of Cry2 by PER1-CRY2
Ki_Cry2_p2c1	2.709023574	Regulation threshold of Cry2 by PER2-CRY1
Ki_Cry2_p2c2	2.309457939	Regulation threshold of Cry2 by PER2-CRY2
Ki_Cry2_rev	0.378297016	Regulation threshold of Cry2 by REV-ERB
Ki_Per1_p1c1	1.010242998	Regulation threshold of Per1 by PER1-CRY1
Ki_Per1_p1c2	1.721720548	Regulation threshold of Per1 by PER1-CRY2
Ki_Per1_p2c1	1.624880971	Regulation threshold of Per1 by PER2-CRY1
Ki_Per1_p2c2	0.368879768	Regulation threshold of Per1 by PER2-CRY2
Ki_Per2_p1c1	1.063344333	Regulation threshold of Per2 by PER1-CRY1
Ki_Per2_p1c2	0.568083313	Regulation threshold of Per2 by PER1-CRY2
Ki_Per2_p2c1	0.86599114	Regulation threshold of Per2 by PER2-CRY1
Ki_Per2_p2c2	0.467627765	Regulation threshold of Per2 by PER2-CRY2
Ki_Rev_p1c1	84.46016203	Regulation threshold of Rev-Erb by PER1-CRY1
Ki_Rev_p1c2	83.99580669	Regulation threshold of Rev-Erb by PER1-CRY2
Ki_Rev_p2c1	84.00271945	Regulation threshold of Rev-Erb by PER2-CRY1
Ki_Rev_p2c2	83.99582755	Regulation threshold of Rev-Erb by PER2-CRY2
Ki_Ror_p1c1	1.094009455	Regulation threshold of Ror by PER1-CRY1
Ki_Ror_p1c2	2.291802041	Regulation threshold of Ror by PER1-CRY2
Ki_Ror_p2c1	0.17734771	Regulation threshold of Ror by PER2-CRY1
Ki_Ror_p2c2	3.087808611	Regulation threshold of Ror by PER2-CRY2
Ki_NHE3_p1c1	0.5248622142	Regulation threshold of NHE3 by PER1-CRY1
Ka_NHE3_p1c2	0.51148790424	Regulation threshold of NHE3 by PER1-CRY2
Ki_NHE3_p2c1	0.04273092406	Regulation threshold of NHE3 by PER2-CRY1
Ki_NHE3_p2c2	0.50842406780	Regulation threshold of NHE3 by PER2-CRY2
Ki_NKCC2_p1c1	7.111624431	Regulation threshold of NKCC2 by PER1-CRY1
Ki_NKCC2_p1c2	6.374145136	Regulation threshold of NKCC2 by PER1-CRY2
Ki_NKCC2_p2c1	6.328221462	Regulation threshold of NKCC2 by PER2-CRY1
Ki_NKCC2_p2c2	0.944237431	Regulation threshold of NKCC2 by PER2-CRY2
Ka_SGLT1_p1c1	5.579866217	Regulation threshold of SGLT1 by PER1-CRY1
Ka_SGLT1_p1c2	0.3368079674	Regulation threshold of SGLT1 by PER1-CRY2
Ka_NCC_p1c1	0.61559096740	Regulation threshold of NCC by PER1-CRY1
Ka_NCC_p1c2	0.17552314947	Regulation threshold of NCC by PER1-CRY2
Ka_ENaC_p1c1	1.0303132234	Regulation threshold of ENaC by PER1-CRY1
Ka_ENaC_p1c2	0.22547393780	Regulation threshold of ENaC by PER1-CRY2

Table A5: Association and dissociation rates (in hour^{-1}) for the protein complexes.

Parameter	Value	Description
kass_cb	0.008965832	CLOCK-BMAL1 association rate
kass_p1c1	0.614435343	PER1-CRY1 association rate
kass_p1c2	3.17074452	PER1-CRY2 association rate
kass_p2c1	0.011242223	PER2-CRY1 association rate
kass_p2c2	0.323930911	PER2-CRY2 association rate
kdiss_cb	0.000499612	CLOCK-BMAL1 dissociation rate
kdiss_p1c1	1.079660064	PER1-CRY1 dissociation rate
kdiss_p1c2	0.016210814	PER1-CRY2 dissociation rate
kdiss_p2c1	1.493335258	PER2-CRY1 dissociation rate
kdiss_p2c2	0.146751896	PER2-CRY2 dissociation rate

Table A6: Translation rates (in hour^{-1}).

Parameter	Value	Description
kp_BMAL1	3.271143875	Bmal1 translation rate
kp_CRY1	0.738295688	Cry1 translation rate
kp_CRY2	0.829860113	Cry2 translation rate
kp_PER1	3.798692975	Per1 translation rate
kp_PER2	1.980575626	Per2 translation rate
kp_REV	0.206633966	Rev-Erb translation rate
kp_ROR	1.875730412	Ror translation rate

Table A7: Maximal transcription rates (in hour^{-1}).

Parameter	Value	Description
V_Bmal1	0.646614483	Bmal1 maximal transcription rates
V_Cry1	0.401023403	Cry1 maximal transcription rates
V_Cry2	0.65185826	Cry2 maximal transcription rates
V_Per1	1.109303016	Per1 maximal transcription rates
V_Per2	1.671549554	Per2 maximal transcription rates
V_Rev	0.006423385	Rev-Erb maximal transcription rates
V_Ror	0.373099089	Ror maximal transcription rates
V_NHE3	2.0096808453	NHE3 maximal transcription rates
V_NKCC2	0.521355199	NKCC2 maximal transcription rates
V_SGLT1	1.0020169907	SGLT1 maximal transcription rates
V_NCC	0.36248470731	NCC maximal transcription rates
V_ENaC	0.52277652371	ENaC maximal transcription rates

A5 Bmal1 knockdown is predicted to reduce CLOCK-BMAL1 oscillation amplitude

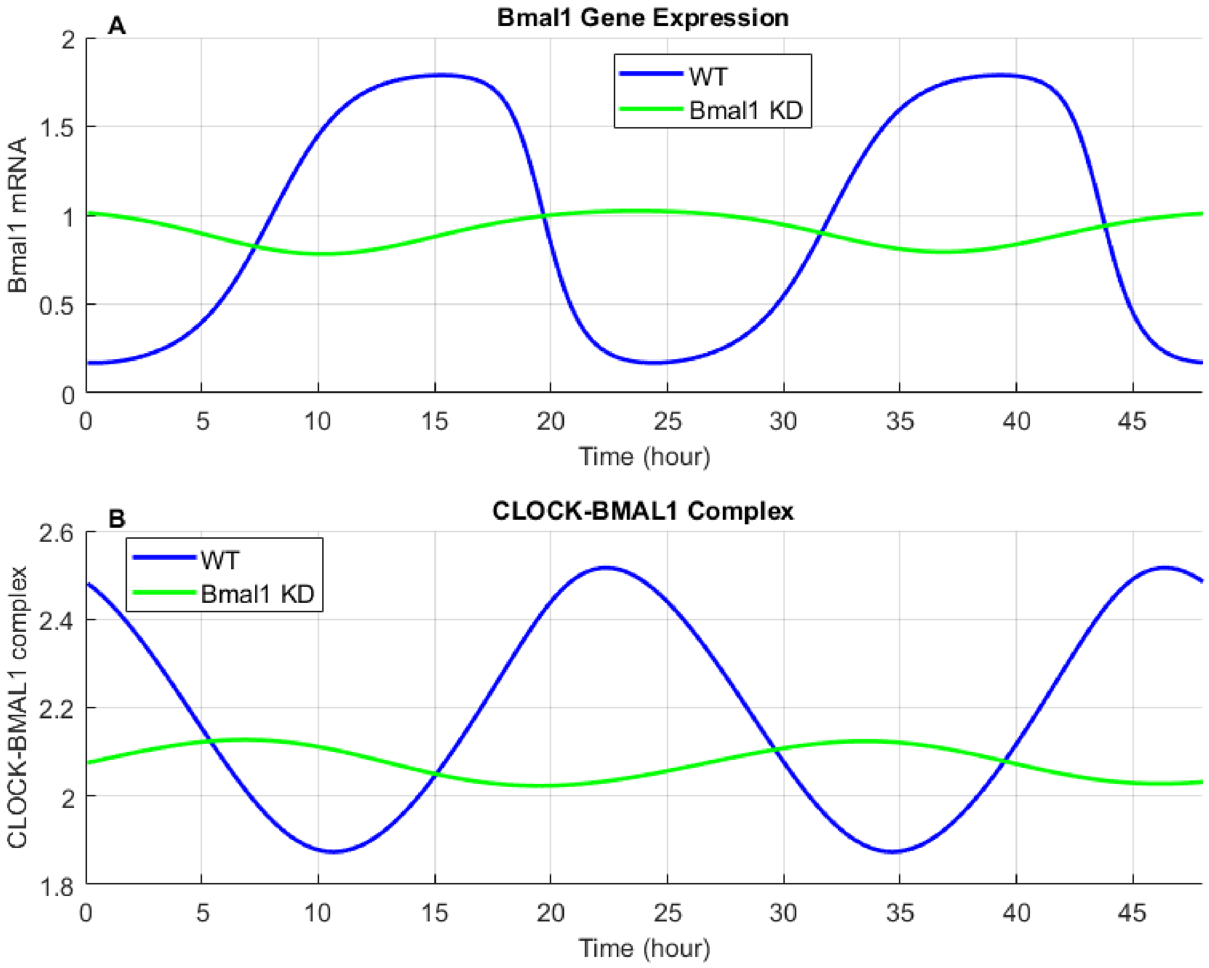

Figure 1: Comparison of simulated circadian gene expression between wild type (WT) and Bmal1 knockdown (KD). Bmal1 KD is simulated by reducing the maximal Bmal1 transcription rate V_{Bmal1} by 40% relative to WT. A: simulated Bmal1 mRNA expression; B: simulated CLOCK-BMAL1 complex level. Both WT and KD simulations were run past transients, and the final 48 h are shown. Time is reported in hours.

As shown in Fig. 1, the mean Bmal1 expression level in the Bmal1 KD simulation is predicted to be 6% lower than in wild type, whereas its oscillation amplitude is reduced to only 15% of the wild-type amplitude. Similarly, the mean CLOCK-BMAL1 complex level in the Bmal1 KD simulation is 5% lower than in wild type, while its oscillation amplitude is reduced to 14% of the wild-type amplitude. Thus, both Bmal1 and the CLOCK-BMAL1 complex show a similar response to Bmal1 KD: a modest decrease in mean level but a substantial attenuation of oscillation amplitude. This is consistent with the model structure, in which CLOCK-BMAL1 complex formation is primarily driven by Bmal1 availability, with CLOCK assumed to be sufficiently available rather than limiting.
